# Global role of the bacterial post-transcriptional regulator CsrA revealed by integrated transcriptomics

**DOI:** 10.1038/s41467-017-01613-1

**Published:** 2017-11-17

**Authors:** Anastasia H. Potts, Christopher A. Vakulskas, Archana Pannuri, Helen Yakhnin, Paul Babitzke, Tony Romeo

**Affiliations:** 1 Department of Microbiology and Cell Science, University of Florida, Institute of Food and Agricultural Sciences, Gainesville, FL 32611-0700 USA; 2 Department of Biochemistry and Molecular Biology, Center for RNA Molecular Biology, Pennsylvania State University, University Park, Pennsylvania, PA 16802 USA; 3 Present Address: Integrated DNA Technologies, Molecular Genetics Department, 1710 Commercial Park, Coralville, IA 52241 USA

## Abstract

CsrA is a post-transcriptional regulatory protein that is widely distributed among bacteria. This protein influences bacterial lifestyle decisions by binding to the 5′ untranslated and/or early coding regions of mRNA targets, causing changes in translation initiation, RNA stability, and/or transcription elongation. Here, we assess the contribution of CsrA to gene expression in *Escherichia coli* on a global scale. UV crosslinking immunoprecipitation and sequencing (CLIP-seq) identify RNAs that interact directly with CsrA in vivo, while ribosome profiling and RNA-seq uncover the impact of CsrA on translation, RNA abundance, and RNA stability. This combination of approaches reveals unprecedented detail about the regulatory role of CsrA, including novel binding targets and physiological roles, such as in envelope function and iron homeostasis. Our findings highlight the integration of CsrA throughout the *E*. *coli* regulatory network, where it orchestrates vast effects on gene expression.

## Abstract (web summary)

The RNA-binding protein CsrA regulates the expression of hundreds of bacterial genes. Here, Potts et al. use several approaches to assess the contribution of CsrA to global gene expression in *E*. *coli*, revealing new binding targets and physiological roles such as in envelope function and iron homeostasis.

## Introduction

Free-living bacteria recognize and adapt to changes in their environment through complex interconnecting regulatory networks^1–3^. Although the *Escherichia coli* transcriptional regulatory networks have been thoroughly studied^4^, the contribution of post-transcriptional regulation to global gene expression patterns even in this model species remains to be clearly understood. Integrated and overlapping transcriptional and post-transcriptional networks mediate complex responses to environmental cues and signals^3, 5^, which are difficult or impossible to predict without detailed knowledge of the circuits. Thus, the relative dearth of information about post-transcriptional regulatory circuitry has hampered the predictive value of known networks. *E*. *coli* possesses many post-transcriptional regulators, including small RNAs (sRNAs)^6^, ribonucleases^7^, RNA helicases^8^, as well as CsrA^9^, Hfq^10^, and other RNA-binding proteins^11^.

CsrA (RsmA) is the key component of the Csr (carbon storage regulator) system, which coordinates numerous complex physiological processes, including motility^9, 12^, carbon metabolism^9, 13, 14^, and virulence of bacterial pathogens^9, 15^. In general, CsrA activates processes that support exponential growth while it represses stationary phase and stress responses, such as biofilm formation and the stringent response^9, 16, 17^. CsrA offers an ideal model for investigating the global influence of bacterial post-transcriptional regulators due to its conserved binding motif, detailed understanding of its regulatory mechanisms, and its broad phylogenetic distribution^9, 18^. CsrA typically binds to sites containing critical GGA motif(s) in the 5′ untranslated region (5′-UTR) and/or the early coding region of its messenger RNA (mRNA) targets, causing changes in RNA structure, translation, stability, and/or transcription elongation^9^. Regulation of CsrA activity is multifactorial, but is mediated largely through molecular mimicry by inhibitory sRNAs, CsrB, and CsrC^9, 16^. Using numerous high-affinity CsrA binding sites, these sRNAs sequester CsrA from its lower affinity mRNA targets. Thus, CsrB/C synthesis and turnover govern expression of the Csr regulon^9, 19^.

Here, we investigate the CsrA regulon of *E*. *coli* during mid-exponential phase growth in LB using next-generation sequencing approaches. UV crosslinking immunoprecipitation and sequencing (CLIP-seq) are used to identify RNAs that interact directly with CsrA in vivo. Ribosome profiling and RNA-seq are used to gage the impact of CsrA on translation, RNA abundance, and RNA stability. For the gene expression studies, we compare a *csrA* wild-type (WT) and *csrA::kan* mutant strain (hereafter *csrA* mutant), which expresses an impaired CsrA protein, with eight-fold lower affinity for RNA^12^. The WT and *csrA* mutant strains grow similarly in rich media, unlike a *csrA* deletion mutant, which grows slowly and rapidly accumulates suppressor mutations^15, 20^. Changes in gene expression caused by this *csrA* mutation likely underestimate the magnitude of CsrA regulatory effects. The integrated application of these approaches provides unprecedented detail of the role of CsrA, revealing vast effects on the expression of structural and regulatory genes throughout the *E*. *coli* regulatory network.

## Results

### CLIP-seq identified hundreds of RNAs that interact with CsrA

**Fig. 1:**
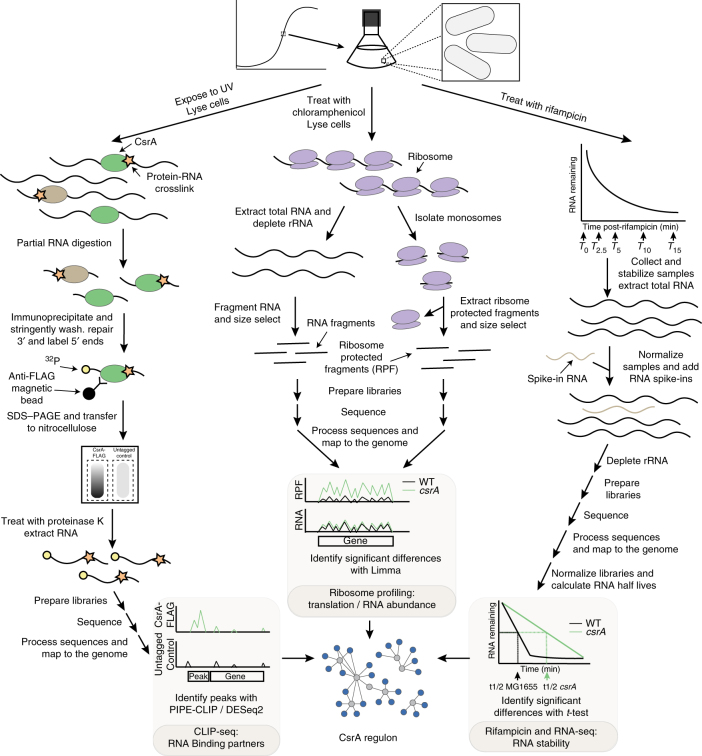
Overview of integrated transcriptomic approaches. Three transcriptomics methods were used to investigate the role of CsrA in gene expression during mid-exponential growth in LB media: UV crosslinking, immunoprecipitation and sequencing (CLIP-seq) (left), ribosome profiling and paired RNA-seq (center), and RNA-seq with rifampicin inhibition of transcription initiation (right). For CLIP-seq analysis, cells were treated with UV light to induce protein–RNA crosslinks, washed, and lysed. The lysate was treated with RNase I to trim the RNA. Then, the crosslinked RNA was co-immunoprecipitated and radiolabeled at the 5′ end. The protein–RNA complexes were separated using SDS–PAGE and transferred to a nitrocellulose membrane. Following proteinase K treatment, the crosslinked RNA was purified, and used to create sequencing libraries. The processed and mapped sequences were used to identify peaks significantly enriched in 3xFLAG-CsrA samples. For ribosome profiling, cells were treated with chloramphenicol to stall transcription elongation and then quickly lysed under conditions that maintain ribosome integrity. Part of the lysate was treated with micrococcal nuclease, and ribosomes were isolated with ultracentrifugation over a sucrose cushion. Ribosome-protected fragments (RPF) were then purified. Total RNA was also extracted from a different portion of the same lysate, depleted of rRNA, and fragmented. RPF and RNA samples were size selected and used to create sequencing libraries. Processed and mapped sequences were analyzed to identify changes in translation (RPF), RNA abundance (RNA), and translational efficiency (RPF and RNA) between the wild-type (WT) and *csrA* mutant strains. For the analysis of RNA stability, samples were collected at five time points following the addition of rifampicin to cultures to prevent transcription initiation. Total RNA was extracted and an RNA spike-in containing exogenous sequences of known abundances was added to aid data normalization. rRNA was depleted and sequencing libraries were prepared. Processed and mapped sequences were normalized using the spike-in RNA reads. Then, RNA stability was modeled using linear regression on a semi-log plot. RNA half-lives were calculated and used to identify significant differences in RNA stability between the WT and *csrA* mutant strains

CLIP-seq has emerged as a reliable method to identify biologically relevant protein–RNA interactions in vivo^10, 21^. It employs UV light to covalently crosslink proteins directly to RNA without crosslinking protein to protein, thus avoiding large covalent complexes common to formaldehyde crosslinking and facilitating the stringent purification of crosslinked RNA. To identify CsrA binding partners in vivo, we conducted CLIP-seq on five biological replicates of both 3xFLAG-CsrA and untagged control strains (Fig. 1). Quality control data are presented in Supplementary Fig. 1a–g. The biological replicates showed high correlations for both untagged and 3xFLAG-CsrA samples (Supplementary Fig. 2a, b). We identified 457 CLIP-seq peaks that were significantly enriched in the 3xFLAG-CsrA samples, including many known CsrA binding partners (Supplementary Figs. 2c, 3a; Supplementary Data 1)^9^. Our CLIP-seq results and previous studies aimed at identifying *E*. *coli* CsrA binding targets on a global scale broadly overlap^17, 22, 23^, supporting the validity of our method (Supplementary Fig. 3b). However, 43% of genes identified with the CLIP-seq analysis were not identified previously in *E*. *coli* (Supplementary Fig. 3b). This variability may arise due to the different experimental procedures that were utilized, namely, CsrA overexpression in previous studies, UV crosslinking here, stringency of washing, and the depth and saturation of sequencing. Growth conditions also varied in these studies.

**Fig. 2:**
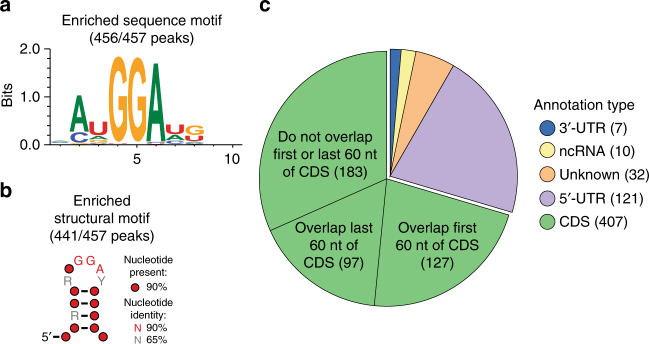
CLIP-seq analysis reveals global patterns of CsrA binding targets. **a** Enriched sequence motif identified in CsrA CLIP-seq peaks with Zagros^70^. **b** Enriched predicted structural motif identified in CLIP-seq peaks with CMFinder^71^ where R = G or A, Y = C or U. **c** Localization of CsrA CLIP-seq peaks relative to known or predicted coding sequences (CDS), ncRNAs^77^, transcriptional start sites^4, 78^, and transcriptional terminators^4^. Each peak may overlap multiple features or types of annotations

Sequence and structural motif analysis identified an AuGGAug motif exposed within a short stem-loop structure as highly enriched in the CsrA CLIP-seq peaks (Fig. 2a, b). This motif was significantly enriched in the CLIP-seq peaks relative to the *E*. *coli* genome sequence (two-sided *χ* ^2^-test, *p* < 0.0001). This is consistent with a previous systematic evolution of ligands by exponential enrichment study, which revealed that both the consensus sequence ruACArGGAugu and GGA sequence localization in the loop of a stem-loop were required for high-affinity binding^24^.

The CsrA CLIP-seq peaks and their associated genomic features are listed in Supplementary Data 1 and summarized in Fig. 2c. Peaks were associated with nine non-coding RNA (ncRNAs). However, the only ncRNA besides CsrB/C whose interaction with CsrA has been previously studied, McaS^25^, was not enriched in our analysis. Seven peaks were identified in 3′-UTRs of mRNAs, something that to our knowledge has not been studied previously. Thus, we confirmed the in vivo data for one of these 3′-UTRs by testing CsrA binding in vitro. The 3′-UTR of *ybjX* was chosen for this analysis because its downstream gene is divergently oriented and there is no evidence in our RNA-seq data for expression of an sRNA from this region (Supplementary Fig. 4a, b). About 21% of the peak annotations overlap 5′-UTRs, and analysis of the relative position of peaks showed enrichment near start but not stop codons (Fig. 2c; Supplementary Fig. 3c, d). Strikingly, the majority of the peaks were found within coding sequences (CDS) of mRNAs (Fig. 2c). This was unexpected, as most of the characterized CsrA targets have binding sites in the 5′-UTR or very early CDS. However, Holmqvist et al.^10^ recently reported a similar distribution of CsrA CLIP-seq peaks in *Salmonella*.

A CsrA binding site located 60 nucleotides (nt) within the *sdiA* coding sequence contributes to CsrA-mediated repression of *sdiA* translation in vivo^26^. We used the position of this site to dissect potential regulatory roles of CsrA CLIP-seq peaks within CDS. There were 127 peaks within CDS that overlap the first 60 nt (Fig. 2c), where CsrA might regulate translation initiation and/or RNA stability similar to known mechanisms^9^. Of the 97 peaks within 60 nt of stop codons, 78 also overlap downstream genes that are in putative operons with the upstream genes (Supplementary Data 1). We hypothesize that some of these sites might be involved in discordant expression within operons, as in *nhaAR* ^27^. The remaining CsrA crosslinking sites are located deep within CDS (Fig. 2c). Recent reports from *Salmonella* ^10^ and *Campylobacter* ^28^ also identified CsrA binding sites deep within mRNA CDS, of uncertain biological relevance. We have found that CLIP-seq peaks within CDS tend to be associated with ribosome pause sites (discussed below).

### CsrA-dependent effects on RNA abundance and translation

Despite the established role of CsrA in regulating translation initiation, until recently there has not been an approach to analyze this on a global scale. Ribosome profiling has emerged as a way to pinpoint the positions of actively translating ribosomes by sequencing the RNA that they protect from nuclease digestion, referred to as ribosome-protected fragments (RPF) (Fig. 1)^29^. We used ribosome profiling and paired RNA-seq to analyze differences in active translation, steady-state RNA abundance, and translational efficiency (TE) between the WT and *csrA* mutant strains. TE is an indicator of post-transcriptional regulation, which is assessed as an interaction term between changes in translation and RNA abundance, i.e., measuring translation while controlling for changes in RNA abundance.

We prepared ribosome profiling and paired RNA-seq libraries from five biological replicates of each strain. The biological replicates showed high correlations for both the RPF and RNA libraries (Supplementary Fig. 5a–d), demonstrating the reproducibility of our approach. We used an integrated model to assess expression, combining tests for changes in translation, RNA abundance, and TE between the *csrA* mutant and WT strains in the 3586 protein-coding genes detected. Translation of 1188 genes, as measured by changes in RPF abundance, was significantly different in WT and *csrA* mutant strains (Supplementary Fig. 6a). The paired RNA libraries identified 1025 genes with significantly altered RNA abundance (Supplementary Fig. 6b). Supplementary Fig. 6d shows the 174 genes whose TE differed between the WT and *csrA* mutant, including genes whose translation initiation was previously reported to be directly controlled by CsrA, such as *nhaR*, *dgcZ*, and *pgaA* ^9, 27^. In addition, many of the genes regulated by CsrA in this analysis were identified recently with a proteomics approach (Supplementary Fig. 7)^23^. We also analyzed CsrA effects on RNA abundance alone for protein-coding genes and non-protein-coding genes. This analysis showed differential expression of 920 genes out of the 3837 detected (Supplementary Fig. 6c). Taken together, these results indicated that CsrA is a global regulator acting primarily as a repressor, and its overall effect on gene expression is negative.

### RNA-seq analysis of the effects of CsrA on RNA stability

CsrA regulates gene expression through effects on RNA stability as a primary effect of CsrA-mRNA binding or as a consequence of its effect on translation initiation^9, 12, 30, 31^. We used RNA-seq to measure RNA abundance at 0, 2.5, 5, 10, and 15 min after transcription initiation arrest with rifampicin. Then, we calculated RNA half-lives for the WT and *csrA* mutant strains (Fig. 1), whose median half-lives were similar, at 3.9 and 3.3 min, respectively. The WT value is close to previously reported average half-lives for *E*. *coli* during exponential growth^32, 33^. Half-lives calculated from RNA-seq and quantitative reverse transcription PCR (qRT-PCR) data were generally well correlated, indicating that our RNA-seq analysis of RNA stability was reliable (Supplementary Fig. 8a). Half-lives from the biological replicates were also well correlated (Supplementary Fig. 8b, c).

Plots of the distribution of RNA half-lives between the two strains showed that many transcripts were destabilized in the *csrA* mutant (Supplementary Fig. 9a, b). This finding was unexpected, given the few examples of RNA stabilization by CsrA^9^ and its overall repression of RNA abundance (Supplementary Fig. 6b, c). However, a recent study proposed that CsrA is a major RNA stabilizing factor in *E*. *coli*, although possible mechanism(s) for this result were not addressed^34^. Altogether, there were 203 RNAs with significantly altered stabilities between the WT and *csrA* mutant strains out of 2564 genes analyzed (Supplementary Fig. 9c), including genes whose RNA stability was previously reported to be controlled by CsrA, such as *dgcZ* ^9^. However, our approach did not permit accurate determination of half-lives for very unstable or low-abundance RNAs. For example, the CsrA-stabilized *flhDC* mRNA was not identified in this analysis, likely due to poor sensitivity in measuring its half-life of <1 min^12^.

### Integrated results from multiple transcriptomics approaches

**Fig. 3:**
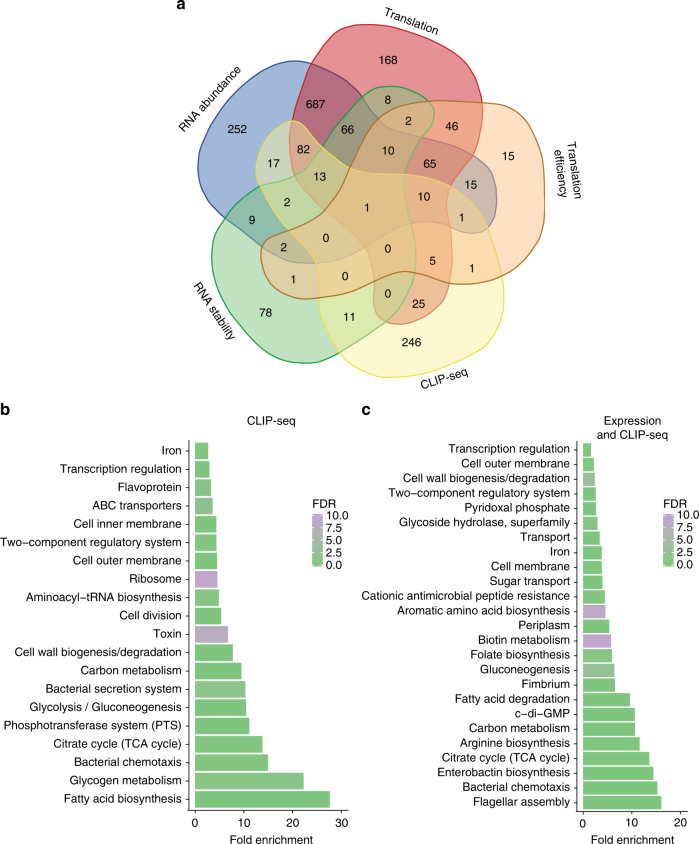
Integration of transcriptomics data. **a** Venn diagram depicting genes with significant changes according to the different transcriptomic approaches. CLIP-seq peaks without associated annotations are not shown. A subset of enriched functional annotations as determined with DAVID^76^ with the full lists in Supplementary Data 3 and 4. **b** Functional annotation of enriched genes that were associated with CsrA CLIP-seq peaks. **c** Functional annotation of differentially expressed genes between the WT and *csrA* mutant strains in any of our transcriptomic approaches or associated with CsrA CLIP-seq peaks. The bars are colored according to the false discovery rate (FDR) of the test for enrichment (EASE test, a modified Fisher’s exact test)

The overlap of genes identified with the different transcriptomics approaches is summarized in Fig. 3a. A large proportion of genes were identified with more than one method, bolstering support for the role of CsrA in their expression. The strongest evidence of direct regulation by CsrA is from genes with associated CLIP-seq peaks and evidence of post-transcriptional regulation, as defined by changes in their TE and/or RNA stability. However, transcription, translation, and RNA stability are often intimately linked in bacteria, such that uncoupling transcription and translation or a decrease in ribosome coverage along a transcript can trigger RNA decay^35^. Fortunately, changes in translation and/or RNA abundance were informative for RNAs that also showed evidence of CsrA binding in the CLIP-seq analysis, even if TE or RNA stability appeared to be unaltered. Supplementary Fig. 10a–e shows genome browser views of genes associated with CLIP-seq peaks with and without evidence of post-transcriptional regulation. One such example is *cstA*, a previously characterized CsrA target (Supplementary Fig. 11). Thus, in this study we defined direct regulatory effects as changes in a gene’s translation, RNA abundance, TE, and/or RNA stability that were associated with a CsrA CLIP-seq peak and indirect effects as those that were not.

The functional significance of CLIP-seq peaks localized in different types of RNA or mRNA segments was explored by examining any associated regulatory trends (Fig. 2c). Consistent with known CsrA regulatory effects, many CLIP-seq peaks overlapping 5′-UTRs were associated with post-transcriptional regulation (15%), primarily via negative effects on TE (Supplementary Data 1, 2)^9^. We found no post-transcriptional regulation for CLIP-seq peaks associated with 3′-UTRs, so further studies are required to determine possible regulatory roles of these sites. Of the CLIP-seq peaks within ncRNAs, only the one overlapping CsrC was associated with changes in RNA stability (Supplementary Data 1, 2), but other possible regulatory roles of these sites are discussed below. Consistent with the hypothesis that CsrA binding at the end of CDS may regulate the expression of downstream genes, few of these CLIP-seq peaks (6%) were associated with post-transcriptional regulation of the upstream gene (Supplementary Data 1, 2). On the other hand, CLIP-seq peaks in the first 60 nt or deep within CDS were more often associated with post-transcriptional regulation (17% and 12%, respectively) (Supplementary Data 1, 2). CLIP-seq peaks overlapping the beginning of CDS were often associated with repression (64%), consistent with many characterized CsrA targets (Supplementary Data 1, 2). In contrast, CLIP-seq peaks located deep within CDS were often associated with CsrA-mediated activation (41%) and most of the post-transcriptional regulation was at the level of RNA stability (Supplementary Data 1, 2). This suggests that CsrA binding within mRNA CDS leads to effects on RNA stability, perhaps by altering nuclease accessibility, similar to its involvement in CsrB decay^36^.

A recent study indicated that CsrA binding entirely within the CDS prevented Rho-dependent transcription termination by stabilizing an alternative mRNA structure^37^. This finding suggests that CsrA binding within CDS can affect RNA structure, with regulatory outcomes. Conceivably, CsrA-dependent changes in RNA structure might affect the translation elongation rate, while ribosome pausing might alter CsrA recognition of binding sites involved in regulation. In either case, CsrA binding within CDS would predominate near translational pause sites. Consistent with this idea, ribosome pause sites were distributed at a significant 1.6-fold enrichment within 30 bases of CsrA CLIP-seq peaks (two-sided *χ* ^2^-test, *p* < 0.0001). Supplementary Fig. 12a, b show a similar bimodal distribution of ribosome pauses associated with CsrA CLIP-seq peaks in the WT and *csrA* mutant strains, although the origin of this pause distribution is not known. There were no differences in the frequency or strength of pauses between the WT and *csrA* mutant strains globally or associated with CLIP-seq peaks, indicating that CsrA does not regulate ribosome pausing (two-sided *t* test, *p* > 0.05). Some examples of CLIP-seq peaks and associated ribosome pauses are shown in Supplementary Fig. 12c–h. Whether abundant CsrA binding within CDS has pervasive regulatory consequences remains to be determined.

Many genes associated with CLIP-seq peaks did not exhibit differential expression in our analyses (Fig. 3a). However, many such peaks overlap known CsrA regulatory targets, such as *hfq* ^30^, *pnp* ^31^, and *dksA* ^17^, and/or contain conserved CsrA binding motifs. These observations suggest that spurious UV crosslinking is unlikely to be the source of such peaks. Some of these CLIP-seq peaks may represent authentic binding sites that do not affect gene expression under the growth conditions used in our analysis. Perhaps their regulation requires additional factors that are unavailable under this condition or is masked by autoregulation, the latter of which was shown previously for *dksA* ^17^. Because the *csrA* mutant in these studies retains partial RNA-binding activity^12^, some transcripts might be regulated in the mutant strain via this residual activity. It is also plausible that some sites do not regulate the target’s expression but instead compete with CsrA binding to regulated targets, similar to CsrB/C or *fimA* mRNA of *Salmonella* ^9^. In this regard, all CsrA binding sites within the transcriptome compete with one another for limiting CsrA^16^. Therefore the global composition and expression level of transcripts would affect the regulatory dynamics of the Csr system.

Functions enriched in genes with CsrA CLIP-seq peaks are shown in Fig. 3b, and genes whose expression was significantly affected in any approach in Fig. 3c. The full results for these categories, as well as genes associated with both CLIP-seq peaks and expression changes are shown in Supplementary Data 3–5. Several of the enriched functional categories represent known roles of CsrA^9^, and a few are explored in more detail in the following sections.

### The role of CsrA in carbon metabolism and transport

**Fig. 4:**
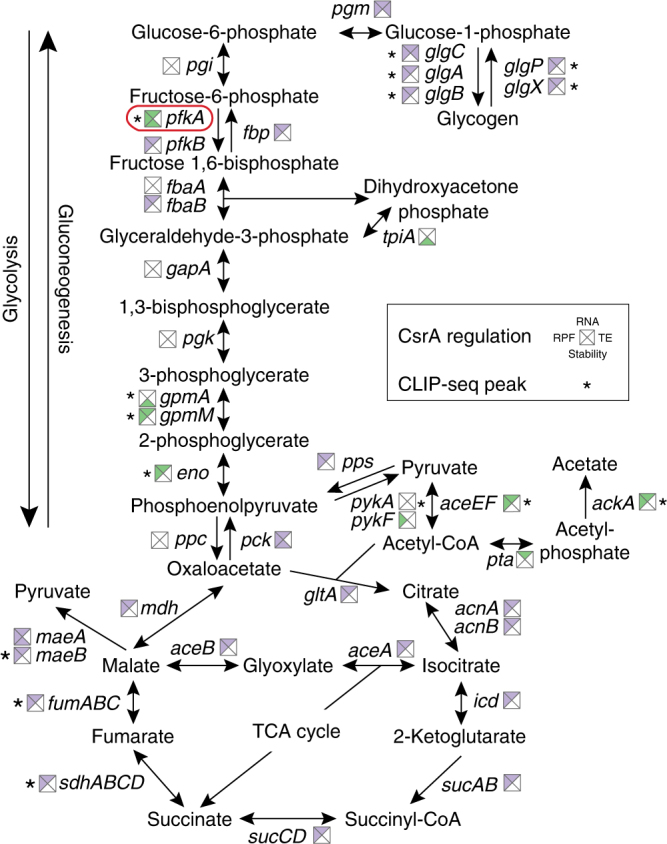
CsrA activates glycolysis and represses gluconeogenesis and the tricarboxylic acid cycle. Effects of CsrA on gene expression in central carbon metabolism. Squares near gene names show effect of CsrA on translation (RPF, left), RNA abundance (RNA, top), RNA stability (stability, bottom), and/or translation efficiency (TE, right). Purple indicates repression by CsrA and green indicates activation. Asterisks indicate that a CsrA CLIP-seq peak is associated with the gene. A red box highlights the key role of *pfkA* on CsrA-dependent effects on glycolysis

CsrA has been known to regulate central carbon metabolism since the 1990s^13^. Recent transcriptomics and metabolomics studies have confirmed that CsrA activates glycolysis, while it represses gluconeogenesis and the TCA cycle^13, 14^. However, the mechanisms used to mediate these changes have remained elusive. Metabolic flux analysis has suggested that phosphofructokinase, encoded by *pfkA*, is key to effects of CsrA on glycolysis^38^, but the molecular basis of this finding remained unclear. We identified a CLIP-seq peak in the 5′-UTR of *pfkA* and this mRNA was destabilized in the *csrA* mutant (Fig. 4). Thus, CsrA binding appears to activate glycolysis by stabilizing *pfkA* mRNA. Additional central carbon metabolism genes exhibited CsrA CLIP-seq peaks, and may be directly regulated by CsrA (Fig. 4). However, the coordinated changes in expression of numerous genes in glycolysis, gluconeogenesis, and the TCA cycle, in the absence of CLIP-seq peaks (Fig. 4), suggests that CsrA may also regulate central carbon metabolism genes indirectly.

Genes involved in the transport of carbon sources were enriched in our transcriptomics data (Fig. 3b, c). For example, CsrA regulates genes encoding several phosphoenolpyruvate-dependent sugar phosphotransferase systems. CsrA represses genes for transport of mannose (*manXYZ*), galactitol (*gatABC*), *N*,*N*′-diacetylchitobiose (*chbBCA*), *N*-acetylglucosamine (*nagE*), and 2-*O*-alpha-mannosyl-d-glycerate (*mngA*), while it activates genes for sorbitol (*srlABE*) and *N*-acetylmuramic acid (*murP*) uptake (Supplementary Data 6). These carbon sources may be important for growth in certain ecological niches. For instance, *N*-acetylglucosamine and mannose, derived from intestinal mucus, support the growth of *E*. *coli* in the mammalian intestine^39^. Overall, CsrA directly or indirectly regulates expression of >200 genes encoding transporters of carbon sources and other nutrients (Supplementary Data 6), indicating that the Csr system plays a major role in governing the ability of *E*. *coli* to capture nutrients from the environment. There was significant overlap between genes regulated by CsrA and cAMP-CRP, the master regulator of carbon catabolite repression (CCR), with regulation often occurring in the opposite direction (Supplementary Fig. 13)^4^. Because glucose activates the turnover of CsrB/C sRNAs^19^ and cAMP-CRP regulates CsrB/C transcription under some conditions^40^, these observations further imply that CsrA participates in CCR. The Csr and cAMP-Crp systems of *Yersinia pseudotuberculosis* also exhibit regulatory interactions^41^, although the circuitry for these interactions are distinct in these two species.

### CsrA regulates genes for maintaining cell envelope integrity

**Fig. 5:**
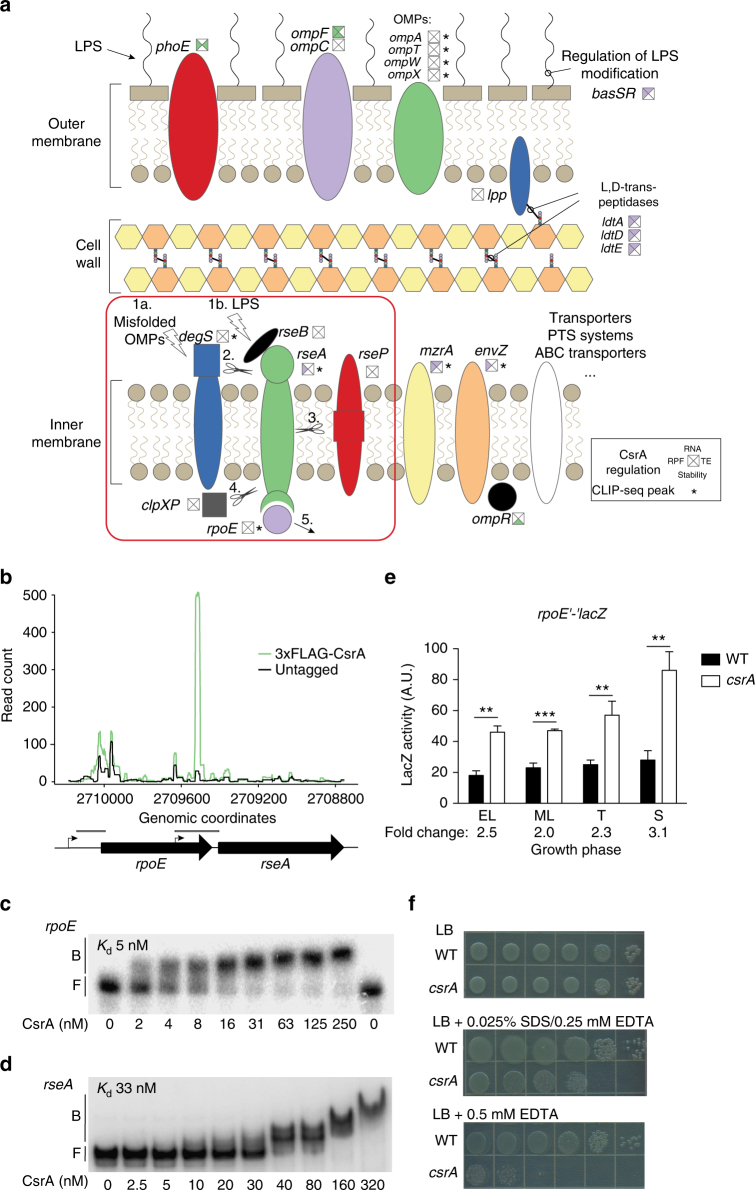
CsrA regulates extracytoplasmic stress responses. **a** Summary of CsrA effects on expression of cell envelope-related genes. The signaling cascade that leads to release of RpoE^79^ is shown surrounded by a red box. The presence of misfolded outer membrane proteins (OMPs) (1a) stimulates DegS proteolytic activity toward RseA (2). LPS also promotes release of RseB to reveal a DegS sensitive site in RseA (1b). After DegS cleavage, RseP cleaves the transmembrane domain of RseA (3). Finally, cytoplasmic ClpXP protease degrades the cytoplasmic portion of RseA (4), thus releasing RpoE from the membrane and allowing it to activate transcription of genes for the envelope stress response (5). Squares near gene names show effect of CsrA on translation (RPF, left), RNA abundance (RNA, top), RNA stability (stability, bottom), and/or translation efficiency (TE, right). Purple indicates repression by CsrA and green indicates activation. Asterisks indicate that CsrA CLIP-seq peak(s) are associated with the gene. **b** 3xFLAG-CsrA and untagged control CLIP-seq read counts mapped to the *rpoE*-*rseA* locus. Position of electrophoretic mobility shift assay (EMSA) probes are indicated by boxes below the plot. **c** EMSA showing of 6xHis-CsrA-*rpoE* RNA interaction (−133 to +19 nt relative to the start codon). Apparent equilibrium binding constant (*K*_d_) is shown. **d** EMSA showing CsrA-*rseA* RNA interaction (−228 to −1 nt relative to the start codon). Apparent *K*_d_ value is shown. **e** β-galactosidase activity of a P_*rpoE*_ *rpoE′*–′*lacZ* translational fusion in wild-type (WT) and *csrA* mutant strains grown in LB at 30 °C. Error bars show standard deviation from the mean of three independent experiments. Two tailed Student’s *t* test was used to determine statistical significance: EL early log (*n* = 3, *p* = 0.006), ML mid-log (*n* = 3, *p* = 0.0002), T transition to stationary (*n* = 3, *p* = 0.0043), and S early stationary phase (*n* = 3, *p* = 0.0017). **f** Growth of serial dilutions of WT and *csrA* mutant strains on LB, LB supplemented with 0.025% SDS/0.25 mM EDTA, and LB supplemented with 0.5 mM EDTA grown overnight at 37 °C. This experiment was repeated twice with similar results

The enrichment analysis revealed that CsrA regulates genes involved in diverse functions related to the cell envelope (Figs. 3b, c, and 5a). Many envelope related genes that were regulated by CsrA are controlled via the extracytoplasmic stress response (ESR) sigma factor, σ^E^ (RpoE), which allows *E*. *coli* to restructure its envelope in response to a variety of stressors^42^. RseA is an inner membrane-bound anti-sigma factor that antagonizes RpoE and is encoded just downstream of *rpoE* (Fig. 5a, b)^42^. RpoE activity is controlled by a complex signaling cascade initiated by sensing of misfolded OM proteins in the periplasm, leading to degradation of RseA and release of RpoE (Fig. 5a). The 5′-UTR of *rseA* exhibited a CsrA CLIP-seq peak, and visual inspection of the RseA CLIP-seq peak in a genome browser also revealed a small peak in the 5′-UTR of *rpoE* though it did not meet our threshold for statistical significance (Supplementary Data 1). To test the hypothesis that CsrA binds to the 5′ UTRs of *rpoE* and *rseA*, we used electrophoretic mobility shift assays (EMSA). We confirmed that CsrA specifically binds to the 5′-UTRs of both *rpoE* and *rseA* in vitro with apparent *K*_d_ values of 5 nM and 33 nM, respectively (Fig. 5c, d; Supplementary Fig. 14a). Translation of *rseA* was repressed by CsrA, but no significant changes in *rpoE* expression were observed (Supplementary Data 2). However, CsrA repressed *rpoE* expression throughout the growth curve as measured using a chromosomal *rpoE′*–′*lacZ* translational fusion grown at 30 °C rather than 37 °C (Fig. 5e). These studies were conducted at 30 °C because this leads to a decreased basal rate of RpoE activation^43^. Thus, CsrA directly represses both *rseA* and *rpoE* expression in vivo, implying that CsrA affects the dynamics of the ESR.

Growth in the presence of EDTA and SDS/EDTA induces extracytoplasmic stress by disrupting the LPS and the OM, and *rpoE* mutants are sensitized to this stress^43^. Figure 5f shows that the *csrA* mutant is sensitized to EDTA and SDS/EDTA when grown at 37 °C. The higher expression of *rseA* in the *csrA* mutant may decrease the release of *rpoE* from the membrane and diminish *rpoE*-mediated control of genes needed for stress resistance. However, sensitivity to this stress can also be increased by changes in the LPS structure, disruption of OM asymmetry, and decreased incorporation of OM proteins^43–45^. We did not find strong effects of CsrA on genes involved in LPS remodeling or biosynthesis, but we observed a marked decrease in expression of *ompF* and *phoE* in the *csrA* mutant (Supplementary Data 2). Although the precise mechanism is unknown, the OM or the response to OM stress is clearly impaired in the *csrA* mutant. The OM of Gram negative bacteria plays an important role as a selective barrier, regulating what reaches the cell membrane^44^. The maintenance of this barrier may be an important role of the Csr system, which to our knowledge has not been previously associated with CsrA homologs.

### CsrA contributes to the regulation of iron metabolism

**Fig. 6:**
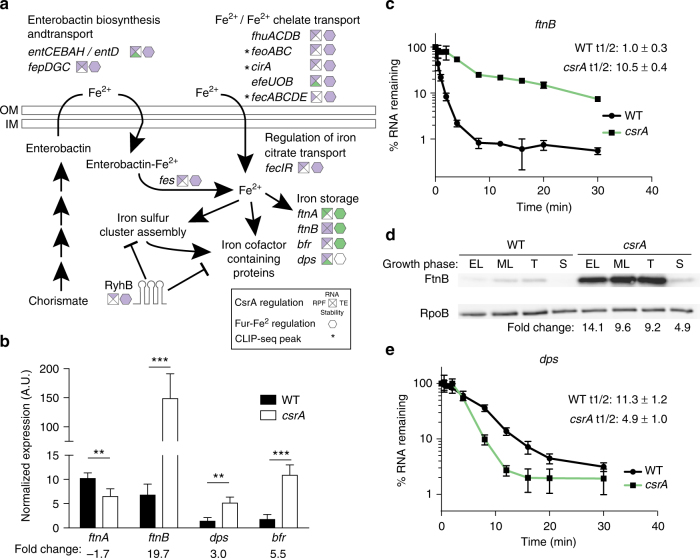
CsrA regulates iron metabolism. **a** Summary of the effects of CsrA and Fur on iron-related genes. Squares near gene names show effect of CsrA on translation (RPF, left), RNA abundance (RNA, top), RNA stability (stability, bottom), and/or translation efficiency (TE, right). Octagons indicate the effects of Fur-Fe^2+^ on RNA abundance adapted from Seo et al.^49^ Purple indicates repression and green indicates activation by CsrA or Fur. Asterisks indicate that CsrA CLIP-seq peak(s) are associated with the gene. **b** qRT-PCR analysis of steady-state *ftnA*, *ftnB*, *dps*, and *bfr* RNA levels normalized to 16s rRNA. Error bars show standard deviation from the mean of three independent experiments. Two tailed Student’s *t* test was used to determine statistical significance: *ftnA* (*n* = 3, *p* = 0.0024), *ftnB* (*n* = 3, *p* = 0.005), *dps* (*n* = 3, *p* = 0.004), and *bfr* (*n* = 3, *p* = 0.0016). **c**, **e** qRT-PCR analysis of *ftnB* (**c**) and *dps* (**e**) RNA stability in wild-type (WT) and *csrA* mutant strains. Error bars show standard deviation from the mean of two independent experiments. Half-life was manually determined from the linear component of the decay curve. Two tailed Student’s *t* test was used to determine statistical significance of the change in half-life: *ftnB* (*n* = 2, *p* = 0.0011), and *dps* (*n* = 2, *p* = 0.0258). **d** Western blot showing 3xFLAG-FtnB levels throughout the growth curve (EL early log, ML mid-log, T transition to stationary phase, and S stationary phase) in WT and *csrA* mutant strains. Full blots are shown in Supplementary Fig. 18. Densitometry was normalized to RpoB levels before calculating fold changes. This experiment was repeated twice with similar results

CsrA affects the expression of numerous genes involved in iron uptake and metabolism, which was reflected in the enrichment analysis in Fig. 3c and summarized in Fig. 6a. CsrA repressed genes involved in iron transport, including those for enterobactin biosynthesis (*entCEBAH* and *entD*), ferrous iron transport (*feoABC* and *efeUOB*), and transport of iron chelates (*fepA*, *fepB*, *fepDGC*, *cirA*, *fecABCDE*, and *fhuACDB*) (Fig. 6a). mRNAs encoding the regulators of *fecABCDE* and therefore iron citrate uptake, FecI, and FecR were also repressed (Fig. 6a). CsrA also decreased the levels of RyhB (Fig. 6a), the sRNA repressor of iron metabolism genes^46^. Many of these effects appear to represent indirect changes in expression, not associated with CLIP-seq peaks (Fig. 6a). Conceivably, these effects may be mediated through Fur, the major regulator of iron homeostasis in *E*. *coli* ^47^. Fur-Fe^2+^ represses genes for siderophore biosynthesis and iron transporters, and it activates iron utilization genes primarily by repressing RyhB expression^47–49^. However, no changes in Fur expression were apparent (Supplementary Data 2). Alternatively, CsrA might mediate these changes indirectly by increasing free intracellular iron pools and thus Fur-Fe^2+^ accumulation.

To this end, we identified several differentially expressed genes involved in iron storage that may affect free intracellular iron accumulation. Although the effect was modest, CsrA activated the expression of *ftnA*, which encodes the major iron storage protein in *E*. *coli* ^50^ (Fig. 6a, b). On the other hand, CsrA strongly repressed the iron storage genes *ftnB*, *dps*, and *bfr* (Fig. 6a, b), which is opposite to the expected effect of Fur-Fe^2+^, an activator of *ftnB* and *bfr* ^49^. CsrA effects on *ftnB* were among the strongest observed in our study (Supplementary Data 2). Additional studies confirmed that CsrA represses *ftnB* mRNA abundance and stability, as well as FtnB protein abundance (Fig. 6b–d). Intriguingly, CsrA repressed *dps* translation and RNA abundance but stabilized its mRNA, which we confirmed with additional qRT-PCR experiments (Fig. 6b, e). These findings suggest complex regulation in which CsrA mediates transcriptional and post-transcriptional effects on *dps* expression in opposing directions. However, we have no evidence that these effects are direct. The protein products of these iron storage genes (FtnA, FtnB, Bfr, and Dps) participate in oxidative stress resistance by sequestering iron^50–52^, donating iron to iron–sulfur clusters damaged by oxidation (FtnB)^52^, and by binding and protecting DNA from oxidizing Fenton chemistry (Dps)^51^. Although no CLIP-seq peaks were associated with these genes, the concurrent increase in *ftnB*, *dps*, and *bfr* expression in the *csrA* mutant may be sufficient to cause a decrease in free intracellular iron. This might lead to an accumulation of apo-Fur in the *csrA* mutant and the observed changes in iron-related gene expression. However, this hypothesis remains to be further explored. Due to the crucial role of iron scavenging during growth and especially during infection^46^, it is conceivable that CsrA effects on iron homeostasis contribute to fitness in the host environment.

### CsrA: a regulator of regulators

**Fig. 7:**
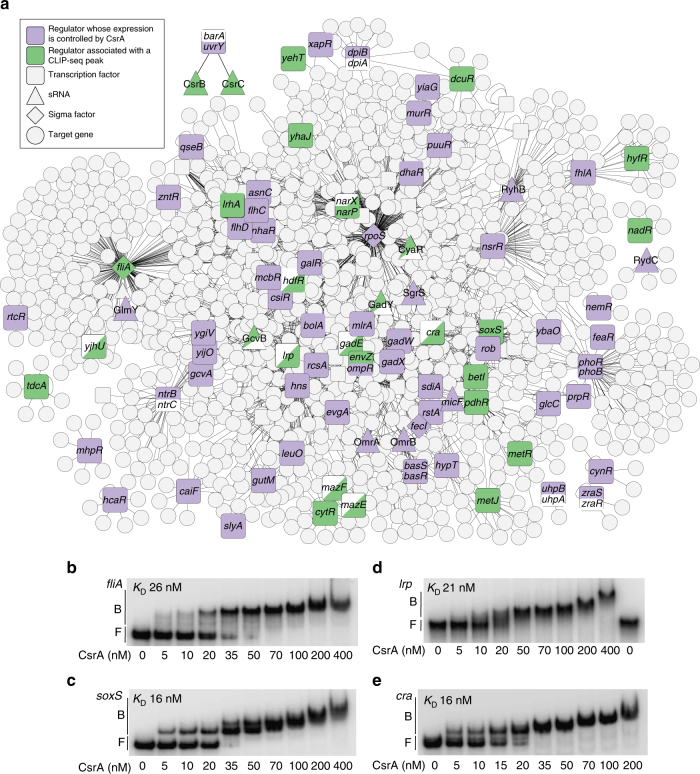
Integration of CsrA into *E*. *coli* regulatory networks. **a** Transcriptional regulators, two-component regulatory system (TCS) sensor kinases, and sRNAs that were differentially expressed in the *csrA* mutant and their target genes are shown. *E*. *coli* transcriptional and sRNA regulatory networks were extracted from RegulonDB^4^. Regulators associated with CLIP-seq peaks and differentially expressed are shown in green and those differentially expressed without an associated CLIP-seq peak are in purple. Those with CLIP-seq peaks that were not differentially expressed are shown in green and white. Regulators represented as squares are transcription factors, triangles are sRNAs, and diamonds are sigma factors. Sensor kinases that were differentially expressed are shown together with their cognate response regulators. **b**–**e** Electrophoretic mobility shift assays (EMSA) showing interaction of CsrA with transcripts of regulatory genes: **b** *fliA* (−30 to +165 nt relative to the start codon). **c** *soxS* (−40 to +87 relative to the start codon), **d** *lrp* (−318 to +45 nt relative to the start codon). **e** *cra* (−81 to +222 nt relative to the start codon)

Because many transcriptional changes occur in the *csrA* mutant without associated CLIP-seq peaks (Fig. 3a), they are likely mediated through other regulators. Indeed, transcriptional regulators and two-component regulatory systems (TCS) are enriched in the CLIP-seq analysis and in CsrA-regulated genes (Fig. 3b, c). CLIP-seq peaks are associated with 46 transcriptional regulators (Supplementary Data 7), including sigma factors, response regulators, ligand-sensing regulators, and autoregulators of toxin–antitoxin (TA) systems. We used EMSA to confirm CsrA interaction with a subset of these mRNAs. CsrA bound specifically and with high affinity to all that were tested, including *fliA*, *soxS*, *lrp*, *cra*, and the intergenic region of the mRNA encoding the PrlF-YhaV TA pair (Fig. 7b–e; Supplementary Figs. 14b–e and 15a–e). Overall, CsrA affected the expression of 87 transcriptional regulators and 11 sensor kinases (Supplementary Data 7). Thus, CsrA appears to have a major impact on the transcriptional landscape of *E*. *coli* both directly and indirectly. In addition, many changes in post-transcriptional regulation were not associated with CLIP-seq peaks (Fig. 3a), suggesting that CsrA may regulate other post-transcriptional regulators. Although CsrA directly represses translation of Hfq and PNPase in *E*. *coli* ^30, 31^, we did not detect significant differences in their expression here (Supplementary Data 2). We suspect that CsrA effects on bulk RNA decay are multifactorial, and found that CsrA regulated several genes encoding RNases (e.g., *rng*, *rnb*, *orn*), perhaps contributing to these effects. We also examined effects on sRNAs as a source of post-transcriptional regulation. CLIP-seq analysis identified peaks overlapping 8 sRNAs, and CsrA affected the abundance of 11 sRNAs (Supplementary Data 7). Except for CsrB and CsrC, there was no overlap between the sRNAs identified with CLIP-seq and those whose abundance was affected by CsrA. An illustration of the transcriptional and sRNA regulators that were differentially expressed in the *csrA* mutant and the genes they regulate reveals extensive integration of CsrA within the regulatory network of *E*. *coli* (Fig. 7a).

**Fig. 8:**
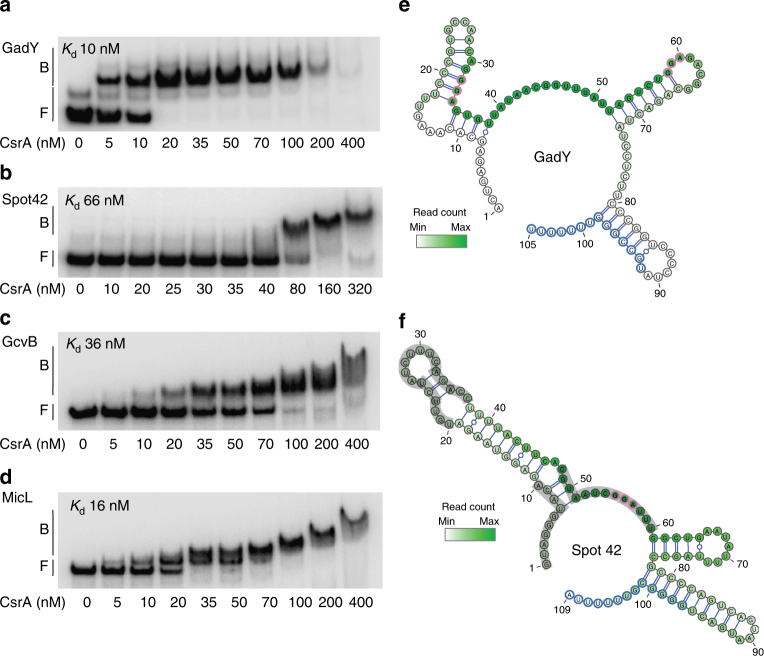
CsrA binds to Hfq-dependent basepairing sRNAs. **a**–**d** Electrophoretic mobility shift assays (EMSA) for the interaction of CsrA with sRNAs. **a** GadY. **b** Spot 42. **c** GcvB. **d** MicL. **e** mFold^80^ predicted GadY structure and **f** Spot 42 structure^55^ with CLIP-seq reads from all five replicates overlaid in green. Putative Hfq binding sites outlined in blue were predicted using the consensus Hfq binding motif identified in *Salmonella* ^10^. GGA motifs are circled in pink. Regions involved in Spot 42 basepairing with mRNA targets are outlined in gray^55^

Beyond the case of McaS^25^, the interaction of CsrA with basepairing sRNAs has not been well studied. Thus, we tested for binding of CsrA to the small basepairing RNAs identified in the CLIP-seq analysis. Direct binding of CsrA to FnrS or CyaR in vitro appeared to be negligible (Supplementary Fig. 16a–d), suggesting that in vivo CsrA interaction with these sRNAs may involve other factors. In contrast, CsrA bound with high affinity and specifically to GadY, Spot 42, GcvB, and MicL (Fig. 8a–d; Supplementary Fig. 14f–i). Closer examination of the CLIP-seq reads within GadY, Spot 42, and GcvB showed significant overlap with known basepairing regions for sRNA–mRNA interaction, which are distinct from where Hfq binds (Fig. 8e, f; Supplementary Fig. 17a). Because GadY is encoded antisense to the *gadX*-*gadW* intergenic target region, its entire length pairs with its mRNA target^53^. Conversely, CLIP-seq reads in MicL lie far upstream of the known basepairing segment of the processed MicL-short product, which accumulates in vivo (Supplementary Fig. 17b)^54^.

For GadY, Spot 42, and GcvB, a reasonable hypothesis is that CsrA binding blocks their ability to pair with target mRNAs. Using our transcriptomics data, we identified examples of CsrA-dependent post-transcriptional regulation of mRNAs that are sRNA targets, which were not associated CLIP-seq peaks (Supplementary Data 8). Most of the sRNA targets were not informative for this analysis, as they did not show post-transcriptional regulation. However, the Spot 42 target genes *galK* and *fucI* were activated by CsrA at the level of TE and RNA stability, respectively. These changes are opposite to known regulatory effects of Spot 42, and are consistent with CsrA-dependent regulation occurring via inhibition of Spot 42 basepairing^55, 56^. CsrA repressed the TE of the GadY target *gadX* (Supplementary Data 8). Although GadY activates *gadX* by stabilizing its RNA, our method may be unable to reliably detect changes in its stability due to its short RNA half-life (~1.5 min)^53^. The mRNA targets of GcvB showed complex responses to CsrA, several of which likely involve direct CsrA binding (Supplementary Data 8). CsrA binding to these sRNAs also raises other hypotheses. CsrA may alter Hfq binding or sRNA stability, which our method would have difficulty assessing due to their small size. The sRNAs might also act as CsrA titrating factors similar to CsrB/C and as proposed for McaS^9^. The latter role was suggested for GadY while this manuscript was under review^57^. As each CsrA dimer contains two symmetric RNA-binding surfaces^9^, it is conceivable that CsrA could interact with an mRNA target and a basepairing sRNA to facilitate their interaction. Further studies are required to elucidate the role of the high-affinity binding of CsrA to these and perhaps other basepairing sRNAs, which were not detectable under our growth conditions.

## Discussion

The use of integrated transcriptomics approaches in this study provides a detailed global view of CsrA effects on gene expression. To our knowledge, this is the first study to measure protein–RNA interactions of a bacterial post-transcriptional regulator concomitantly with its effects on RNA stability, translation, and RNA abundance on a global scale. Our findings indicate that CsrA governs the expression of a vast number of genes, similar in magnitude to global regulatory transcription factors, such as RpoS^58^, DksA^59^, Lrp^60^, and CRP^61^. This study highlights the important role of post-transcriptional regulation in general, and the Csr system in particular, in regulating bacterial gene expression. The diverse effects of CsrA observed here demonstrate that the Csr system plays a major role in determining bacterial lifestyle decisions that require sweeping reallocation of genetic and metabolic resources^16^. Guided by the transcriptomics results, we also explored new roles of CsrA in a limited number of important cellular processes. Beyond the scope of the present investigation, our data provide an abundant resource for further investigation of bacterial regulatory networks, RNA regulatory mechanisms, physiological processes, and metabolic pathways.

## Methods

### Bacterial strains and culture conditions

Bacterial strains used in this study are in listed in Supplementary Data 9. Strains were grown in LB medium (1% tryptone, 1% NaCl, and 0.5% yeast extract) supplemented with kanamycin (50 μg mL^−1^) or gentamycin (10 μg mL^−1^) when appropriate. Glycerol stocks were used to inoculate 2.5 mL of LB, and cultures were grown overnight at 37 °C with shaking at 250 rpm. Fresh media were inoculated from these overnight cultures to an OD_600_ of 0.01, and samples were collected at OD_600_ of 0.2 (early exponential phase), 0.5 (mid-exponential phase), 1.0 (transition to stationary phase), and ~4 (stationary phase), depending on the experiment. FLAG-tagged proteins were created using the λ red recombinase method, as described previously^62^.

### Oligonucleotides and sequencing library mapping statistics

Oligonucleotides synthesized for use in this study are listed in Supplementary Data 10. A summary of sequencing read data is in Supplementary Data 11.

### CLIP-seq

UV crosslinking and immunoprecipitation method was adapted from Huppertz et al.^63^ and Holmqvist et al.^10^ with modifications. Five biological replicates of 3xFLAG-CsrA and WT untagged control strains were grown to mid-exponential phase in 22 mL LB. Cells in the growth medium were then exposed to 400 mJ of UV at 254 nm, washed with PBS, and snap frozen in liquid nitrogen. Samples were thawed, resuspended in 2 mL of RIPA buffer (25 mM Tris-HCl pH 7.6, 150 mM NaCl, 1% NP-40, 0.5% sodium deoxycholate, and 0.1% SDS) with an EDTA-free protease inhibitor cocktail (Roche), and lysed using three rounds of sonication at 0.5 s on/0.5 s off for 10 s on ice. Lysates were cleared by centrifugation then treated with Turbo DNase (Ambion) and RNAse I (Ambion) at 37 °C for 5 min. Samples were treated with anti-FLAG M2 paramagnetic particles (Sigma M8823) to immunprecipitate CsrA for 4 h at 4 °C. The paramagnetic particles were washed three times with high-salt wash buffer (RIPA with 1 M NaCl), once with RIPA, and once with T4 polynucleotide kinase buffer (NEB). Samples were then radiolabeled with [γ^32^P]ATP using T4 polynucleotide kinase (NEB). Protein–RNA complexes were eluted from the beads with Laemmli buffer without β-mercaptoethanol, separated by 12% Bis Tris SDS–PAGE, and transferred to Protran 0.2 μm nitrocellulose membranes. RNA was eluted from the membrane by treatment with proteinase K (Roche). RNA was then purified with phenol-chloroform extraction and ethanol precipitation. The RNA was then depleted of ribosomal RNA (rRNA) with the Ribo-Zero rRNA Removal Kit for Gram Negative Bacteria (Epicentre), according to the manufacturer’s instructions. Sequencing libraries were generated with the NEBNext Small RNA Library Prep Kit (NEB), according to the manufacturer’s instructions with one modification. Adaptors and reverse transcription primers were diluted 1 in 5 in water before use. Libraries were sequenced using HiSeq 2500 50SE (Illumina) by the HudsonAlpha Genomic Services Laboratory.

Sequencing reads were demultiplexed and trimmed with cutadapt^64^ to remove adaptor sequences. Reads longer than 12 bases were then mapped to *E*. *coli* rRNA sequences with bowtie^65^ and unmapped reads were retained. The rRNA depleted reads were then mapped to the *E*. *coli* genome. Integrated genome viewer was used to visualize the alignments^66^. Duplicate reads were removed using Picard MarkDuplicates. The 3xFLAG-CsrA reads were merged into a single file and used as input into PIPE-CLIP^67^ to identify CLIP-seq peaks. These peaks were used to count reads per peak with HTseq count^68^ for each individual sample file. DESeq2^69^ was used to identify peaks significantly enriched (adjusted *p*-value < 0.05) in the 3xFLAG-CsrA samples over the untagged controls. The average length of the CLIP-reads was 17 bases and the significantly enriched CLIP-seq peaks was 136 bases.

### Structural and sequence motif enrichment analysis

Zagros was used to identify enriched sequence motifs in the CLIP-seq peaks using both secondary structure and sequence information^70^. CMfinder was used with default settings to identify the top enriched structural motif^71^. To compare the frequency of the AuGGAug in the CLIP-seq peaks to the *E*. *coli* genome, FIMO (find individual motif occurrences)^72^ software was used to identify this motif in both sets of sequences. A two-sided *χ* ^2^-test was used to determine statistical significance of the enrichment of this sequence in CLIP-seq peaks.

### Ribosome profiling

This method was adapted from Becker et al.^73^ and Ingolia et al.^21^ with modifications. Briefly, five biological replicates of WT and *csrA* mutant cultures were growth to mid-exponential phase in 500 mL of LB. Cultures were treated with 100 μg mL^−1^ chloramphenicol for 5 min at 37 °C with shaking at 250 rpm then quick chilled with an equal volume of ice containing 100 μg mL^−1^ of chloramphenicol. Cells were washed with ice-cold wash buffer (20 mM Tris-HCl pH 7.4, 10 mM MgCl_2_, 50 mM NH_4_Cl, and 1 mM chloramphenicol) and snap frozen in liquid nitrogen. Thawed cell pellets were resuspended in lysis buffer (wash buffer plus 5 mM CaCl_2_, 0.5 mM DTT, 0.4% TritonX-100, and 0.1% NP-40) and lysed by bead beating using 0.1 mm zirconium beads. Samples were vortexed for a total of 8 min in 1.5 increments. Lysate was centrifuged at 4 °C for 30 min at 13,000 rpm to remove cell debris in a tabletop microcentrifuge. Total RNA was extracted from lysates using the miRNeasy Kit (Qiagen), treated with Turbo DNase (Ambion), and purified with the RNeasy Kit (Qiagen). Lysates were diluted to contain an estimated 1.5 mg of RNA and were treated with 1000 U of micrococcal nuclease for 1 h at 25 °C then quenched with EGTA. This treated lysate was ultracentrifuged over a sucrose cushion (wash buffer plus 1 M sucrose) for 4 h at 72,000 rpm (243,275×*g*) at 4 °C in an MLA-130 rotor. RPF were then extracted from the pelleted ribosomes with the miRNeasy Kit (Qiagen). Sequencing libraries were prepared for RPF and RNA samples as described in Becker et al.^73^ except for using the PCR primers listed in Supplementary Data 10. Samples were sequenced using SE50 HiSeq 2000 (Illumina) by the HudsonAlpha Genomic Services Laboratory.

Sequencing reads were trimmed with cutadapt^64^ to remove adaptor sequence from the 3′ end and the first base at the 5′ end that often appeared to be a result of untemplated base addition by reverse transcriptase. Sequences longer than 12 bases were mapped to *E*. *coli* rRNA sequences with bowtie^65^ and unmapped reads were retained. The rRNA depleted reads were then mapped to the *E*. *coli* genome and read counts per gene calculated with HTSeq count^68^. Integrated genome viewer was used to visualize the alignments^66^.

For the integrated analysis of differential translation, RNA abundance, and TE, non-protein-coding genes were removed. Genes with expression lower than an average of 15 counts per sample in both RPF and RNA samples were also removed. The remaining 3586 protein-coding genes were analyzed using limma^74^. A linear model consisting of three contrasts was fitted to the data to identify genes with changes in translation $\left( {{\mathrm{RPF}}_{csrA} - {\mathrm{RPF}}_{{\mathrm{WT}}}} \right)$, RNA abundance (${\mathrm{RNA}}_{csrA} - {\mathrm{RNA}}_{{\mathrm{WT}}}$), and their interaction, TE ($\left( {{\mathrm{RPF}}_{csrA} - {\mathrm{RPF}}_{{\mathrm{WT}}}} \right) - ({\mathrm{RNA}}_{csrA} - {\mathrm{RNA}}_{{\mathrm{WT}}})$). A gene level multiple testing correction was done using a nested F test, which identified the contrasts that contributed significantly to gene level differences. Contrasts with significant F tests and fold changes larger than ±log_2_(0.5) were considered be significant. To identify changes in RNA abundances for both protein-coding and non-protein-coding genes, all genes with an average of 15 counts per RNA sample were also analyzed separately using limma^74^. The linear model (RNA_*csrA*_ − RNA_WT_) was fit to the data to identify genes with differences in their RNA abundance. False discovery rate (FDR) of 5% was used to adjust for multiple testing. Differences were considered significant when the adjusted *p*-value was <0.05 and fold changes were larger than ±log_2_(0.5).

Analysis of ribosome pausing was carried out as previously described^75^, with a few modifications. The first and last 30 nt of CDS were not included to avoid considering initiating or terminating ribosomes as pauses. Positions with >10-fold occupancy over the average occupancy of each CDS were considered to represent pauses. Only pauses shared across at least four biological replicates were considered significant. The frequency of pause sites within trimmed CDS and CLIP-seq peaks ±30 flanking bases was calculated. A two-sided *χ* ^2^-test was used to assess the statistical significance of the difference in the frequency of pausing. The strength of pausing was also compared between WT and *csrA* mutant strains using the fold occupancy over the average occupancy of the trimmed CDS for all pauses and pauses associated with CLIP-seq peaks.

### RNA-seq analysis of RNA stability

RNA decay curves were conducted as previously described^8^. WT and *csrA* mutant strains were grown to mid-exponential phase in LB at which time rifampicin was added to a final concentration of 200 μg mL^−1^ to arrest transcription initiation. Samples were collected 0, 2.5, 5, 10, and 15 min after rifampicin addition and mixed with 2 vol of RNA Protect Bacteria (Qiagen) to immediately stabilize the RNA. The RNA was purified according to the manufacturer’s instructions with the miRNeasy Kit (Qiagen), treated with turbo DNAase I (Ambion), and purified with the RNeasy Kit (Qiagen). RNA concentrations were normalized and ERCC RNA spike-ins (Ambion) were added to represent 5% of the total RNA. rRNA was then depleted using the Ribo-Zero rRNA Removal Kit for Gram negative bacteria (Epicentre). Sequencing libraries were then constructed and sequenced using HiSeq 2000 PE100 (Illumina) by BGI America.

Sequencing reads were demultiplexed, mapped to the *E*. *coli* genome and the ERCC spike-in RNA sequences with bowtie^65^, and read counts per gene determined using HTseq count^68^. Each time point (*T*_n_) was scaled relative to the time zero (*T*_0_) within its time course to normalize the raw read counts. Normalization factors, *k*, were calculated using each exogenous RNA in the ERCC spike-in mix with $k = (\frac{{{\mathrm{ERCC}}_{\mathrm{n}}}}{{{\mathrm{ERCC}}_0}})/(\frac{{{\mathrm{Total}}_{\mathrm{n}}}}{{{\mathrm{Total}}_0}})$, where ERCC_n_ and ERCC_0_ are the total reads for this RNA in library *T*_n_ and *T*_0_, respectively; and Total_n_ and Total_0_ are the total library sizes in library *T*_n_ and *T*_0_, respectively. *k* values were calculated for the 25 most abundant RNAs in the ERCC spike-in, and the geometric average of these was used as the library normalization factor. Raw read counts were divided by the appropriate normalization factor to produce normalized read counts. Curves were fit to the normalized data with linear regression on a semi-log plot with Prism 6 (Graphpad). Genes with starting read counts <50 and *R* ^2^ values <0.7 were excluded from further analysis. Half-lives were calculated for the remaining 2564 genes with the equation $t_{1/2} = - \frac{{{\mathrm{ln2}}}}{{{\mathrm{slope}}}}$. Differences in RNA half-lives between WT and *csrA* mutant strains were analyzed with Student’s two tailed *t* tests and adjusted for multiple comparisons using a FDR of 5%. Genes with FDR < 0.05 and fold changes >log_2_ (0.5) were considered to have significant changes in their RNA stability.

### Functional enrichment analysis

Genes identified in the CLIP-seq analysis and genes whose expression was regulated by *csrA* were analyzed with DAVID to identify clusters of functional terms^76^. The default parameters and databases were used and multiple testing adjustments were done with a FDR cut off of 10%.

### Electrophoretic gel mobility shift assays for RNA binding

Binding of CsrA to RNAs was determined by EMSA with recombinant CsrA-His_6_^17^ and RNA synthesized in vitro with MEGAshortscript Kit (Ambion) or the Ampliscribe T7 flash Transcription Kit (EpiCentre). The template DNA for in vitro transcription of sRNAs was generated by PCR from MG1655 genomic DNA, using the primers listed in Supplementary Data 10. In vitro-transcribed sRNAs were gel-purified, treated with Antarctic phosphatase (NEB), and radiolabeled at the 5′ end using [γ-^32^P] ATP and T4 polynucleotide kinase. Binding reactions contained 0.1–0.5 nM RNA, 10 mM MgCl_2_, 100 mM KCl, 32.5 ng total yeast RNA, 20 mM DTT, 7.5% glycerol, 4 U SUPERasin (Ambion), and various concentrations of CsrA (0–400 nM), and incubated at 37 °C for 30 min. Reactions mixtures were separated on native polyacrylamide gels using 1× TBE as the electrophoresis buffer and labeled RNA was analyzed using a PMI phosphorimager (Bio-Rad) and Quantity One software (Bio-Rad), as described previously^17^. Apparent equilibrium binding constants (*K*_d_) presented in the figures are averages of two independent experiments.

### qRT-PCR

qRT-PCR was used to analyze RNA abundance with an iCycler thermocycler (Bio-Rad Laboratories) and iScript One-Step RT-PCR Kit with SYBR Green (Bio-Rad), according to the manufacturer’s instructions, as described previously^8^. The primers used are listed in Supplementary Data 10. Melting curve analysis was used to verify the specificity of the PCR reaction. Melt curves were generated via denaturation at 95 °C for 1 min followed by lowered to 55 °C for 1 min, and followed by ramping at 0.5 °C/10 s cycles until 95 °C. RNA concentrations were determined with technical duplicates relative to a standard curve using the iQ5 software (Bio-Rad).

### Western blotting

Western blotting was conducted as previously described^8^. Cultures were harvested by centrifugation, resuspended in Laemmli sample buffer, and lysed by boiling for 5 min at 95 °C followed by sonication. Samples were separated using Bis Tris SDS–PAGE with a Criterion cell (Bio-Rad) and electroblotted onto 0.2 μm polyvinylidene difluoride membranes with a Criterion blotter (Bio-Rad). Membranes were blocked with SuperBlock T20 (Thermo Fisher) and probed with Anti-FLAG M2 monoclonal antibody (1:5000 dilution, Sigma) or anti-RpoB monoclonal antibody (1:10,000 dilution, Neoclone). Signal was detected with horseradish peroxidase-linked anti-mouse secondary antibody (GE Healthcare) and SuperSignal West Femto Chemiluminescent Substrate (1:50,000 dilution, Thermo Fisher). Blots were imaged using the ChemiDoc XRS+ system (Bio-Rad), and signals were quantified using Quantity One image analysis software (Bio-Rad). The western blots and fold change values shown are representative from two independent experiments.

### LacZ fusion strain construction and β-galactosidase assay

The *rpoE′–′lacZ* fusion strain was constructed using the conditional-replication, integration, and modular (CRIM) system, as previously described^26^. The fusion contains from −292 to +26 relative to the *rpoE* translation initiation codon. β-galactosidase experiments were performed as described previously^26^.

### Envelope stress plating

WT and *csrA* mutant strains were grown in LB at 37 °C with shaking at 250 rpm to mid-exponential phase, washed with PBS, serially diluted, and plated on LB alone or LB supplemented with EDTA and/or SDS. Plates were incubated at 37 °C for 18 h then colonies were counted.

### Statistical analysis

Statistical analysis was carried out in the R computing environment and in Prism 6 (Graphpad). Relevant statistical information is included in the methods for each experiment. Error bars show standard deviation from the mean. Asterisk representing statistical significance denote the following *p < 0.05, **p < 0.005, ***p < 0.0005.

### Data availability

The CLIP-seq, ribosome profiling, and RNA-seq raw sequencing data have been deposited in the GEO repository with accession code GSE102386. All other relevant data are available in this article and its Supplementary Information files.

## Electronic supplementary material

Supplementary Information

Description of Additional Supplementary Files

Supplementary Data 1

Supplementary Data 2

Supplementary Data 3

Supplementary Data 4

Supplementary Data 5

Supplementary Data 6

Supplementary Data 7

Supplementary Data 8

Supplementary Data 9

Supplementary Data 10

Supplementary Data 11
