# Supplementary material for: Global role of the bacterial post-transcriptional regulator CsrA revealed by integrated transcriptomics
Source: Nat Commun. 2017 Nov 17;8:1596. doi: 10.1038/s41467-017-01613-1 (PMC5694010; doi:10.1038/s41467-017-01613-1)
Supplement: Supplementary file 1 — Supplementary Information [file 41467_2017_1613_MOESM1_ESM.pdf]

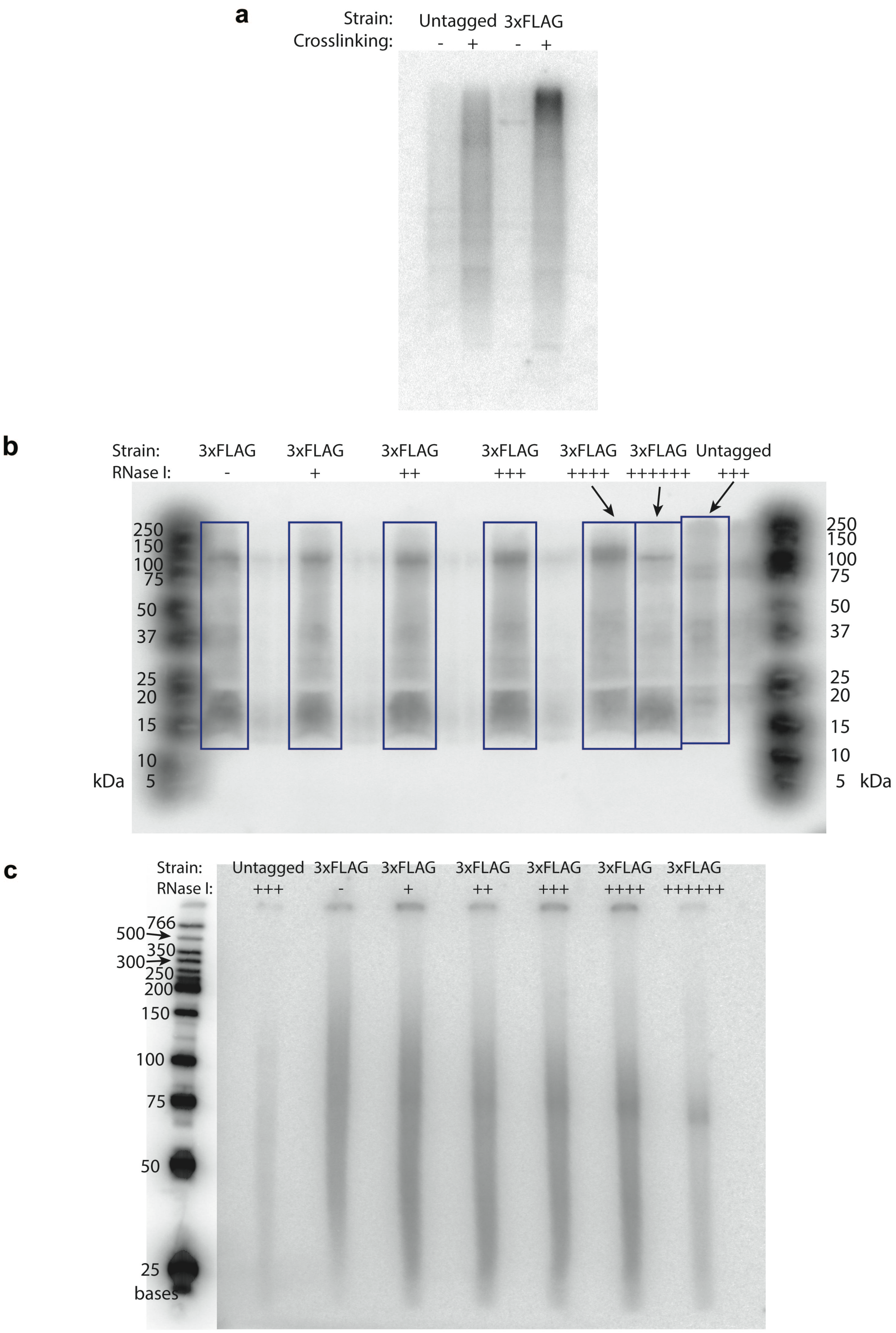
a
Strain: Untagged 3xFLAG
Crosslinking: - + - +
b
Strain: 3xFLAG 3xFLAG 3xFLAG 3xFLAG 3xFLAG 3xFLAG Untagged
RNase I: - + ++ +++ ++++ ++++++ +++
250 150 100 75 50 37 25 20 15 10 5
kDa
c
Strain: Untagged 3xFLAG 3xFLAG 3xFLAG 3xFLAG 3xFLAG 3xFLAG
RNase I: +++ - + ++ +++ ++++ ++++++
766 500 350 300 250 200 150 100 75 50 25
bases

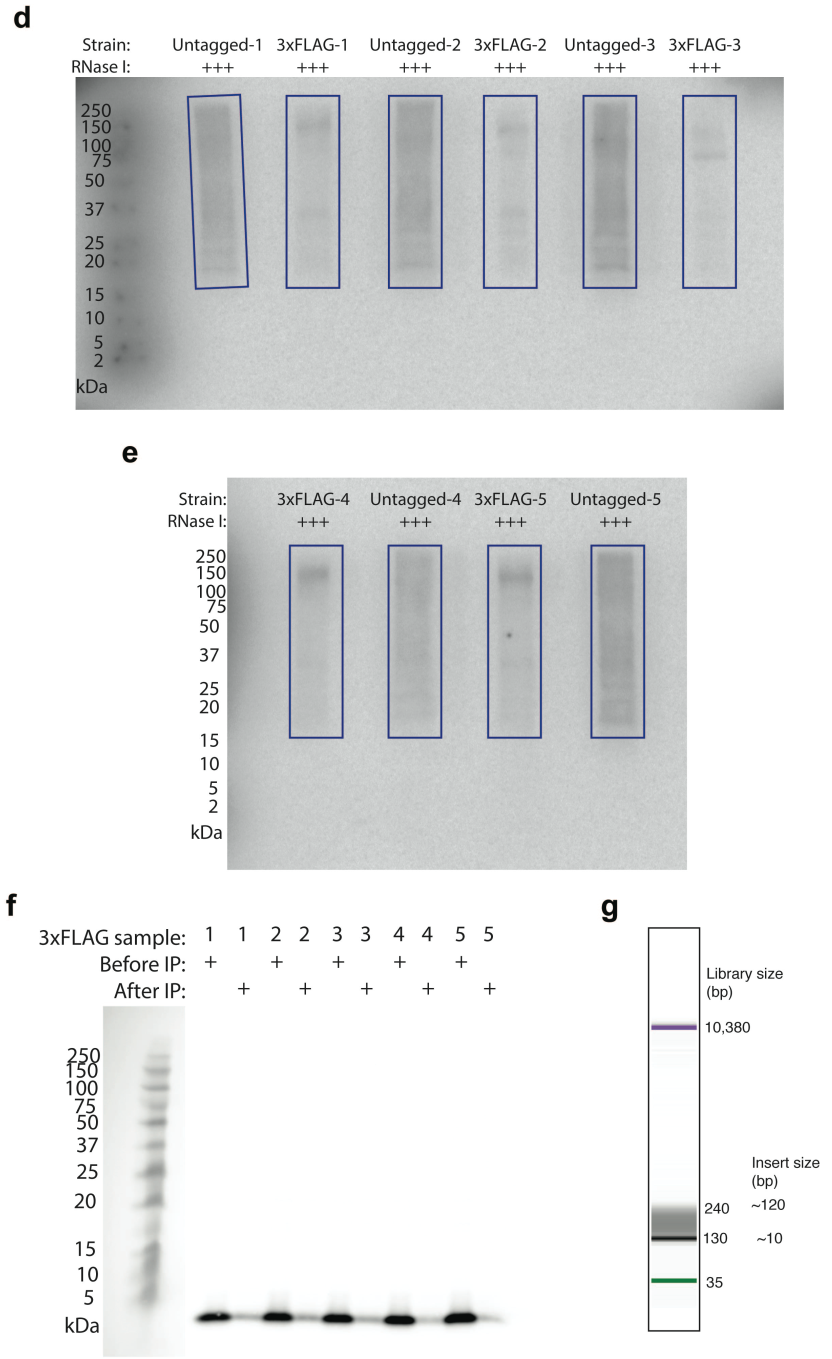

Supplementary Figure 1: CLIP-seq protein-RNA crosslinking optimization
a. Radiograph of nitrocellulose membrane containing immunoprecipitated 3xFLAG-CsrA and untagged control strains with and without crosslinking. Samples were prepared as described in the Materials and Methods, except non-crosslinked samples were not exposed to UV. b. Radiograph of nitrocellulose membrane containing immunoprecipitated 3xFLAG-CsrA and untagged control

strains with increasing concentrations of RNase I. Samples were prepared as described in the Materials and Methods, except the variation of RNase I concentration. Blue boxes show the region cut out of the membrane, which was then treated with proteinase K and the crosslinked RNA extracted and purified (see Materials and Methods). Dye conjugated protein standards (5-250 kDa) were marked with γ-^{32}P ATP to allow visualization in radiograph. c. Radiograph of purified crosslinked RNA from b and radiolabeled DNA standards. Samples and standards were denatured then separated on denaturing TBE-PAGE. Based on these data, we chose the RNAse I (Life Technologies) concentration denoted as +++ (800 units), as it yielded a distribution of RNAs from 25-125 bases. d-e. Radiographs of nitrocellulose membrane containing immunoprecipitated 3xFLAG-CsrA and untagged control strains used for library construction. The exposure time for these image were shorter than in a. f. Western blot of 3xFLAG-CsrA lysate before and after removal of 3xFLAG-CsrA-RNA complexes by immunoprecipitation with M2 anti-FLAG paramagnetic particles (Sigma) using samples from d-e. Note that the incidence of crosslinking is extremely low and crosslinked samples are not visible in Western blots. g. Example Bioanalyzer gel image of a final library that was sized selected to include insert sizes roughly similar to sizes expected based on results in b of ~10-120 bases.

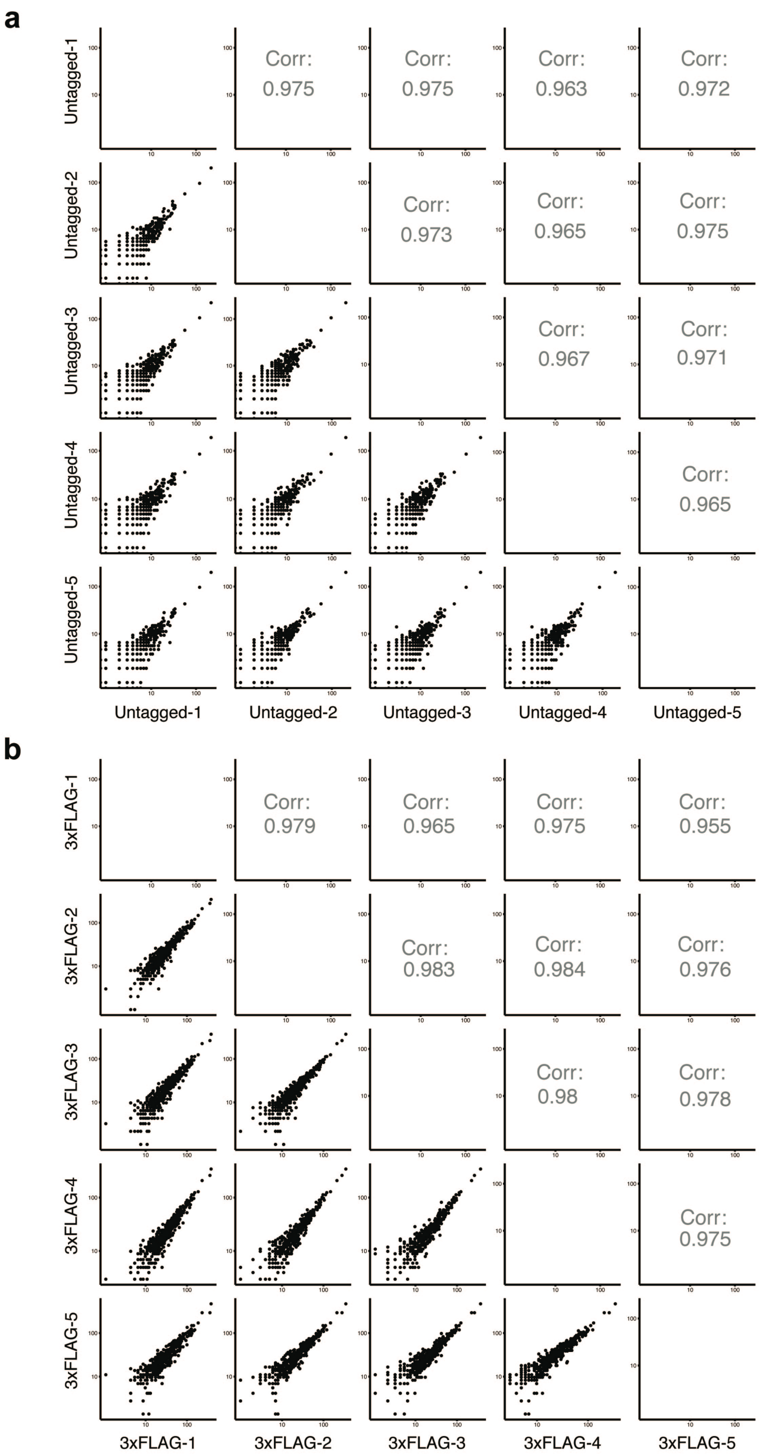

a
Untagged-1
Untagged-2
Untagged-3
Untagged-4
Untagged-5
Corr: 0.975
Corr: 0.975
Corr: 0.963
Corr: 0.972
Corr: 0.973
Corr: 0.965
Corr: 0.975
Corr: 0.967
Corr: 0.971
Corr: 0.965
b
3xFLAG-1
3xFLAG-2
3xFLAG-3
3xFLAG-4
3xFLAG-5
Corr: 0.979
Corr: 0.965
Corr: 0.975
Corr: 0.955
Corr: 0.983
Corr: 0.984
Corr: 0.976
Corr: 0.98
Corr: 0.978
Corr: 0.975

c

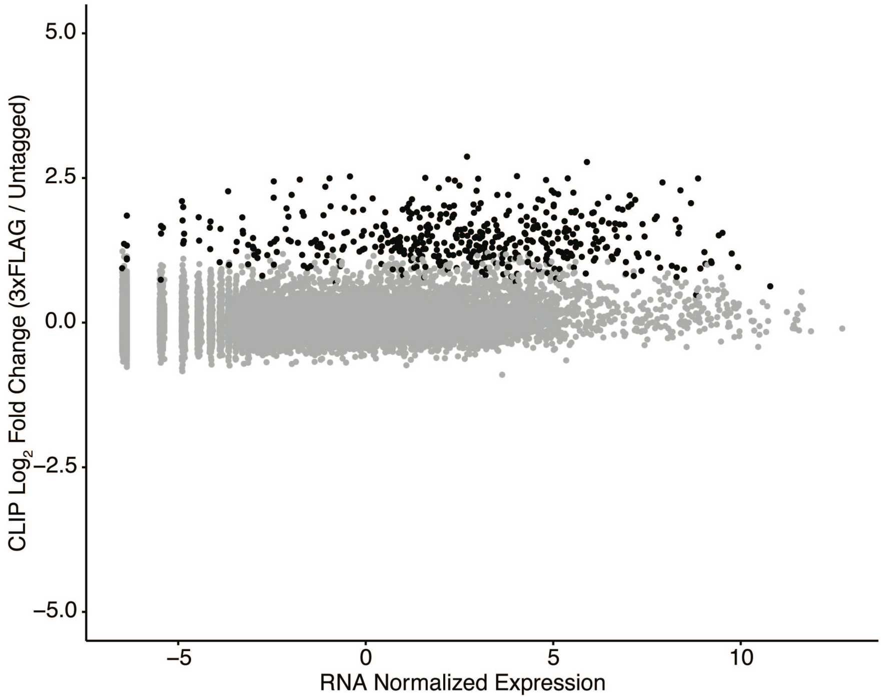

Supplementary Figure 2: CLIP-seq peaks are reproducible and not dependent on expression levels

Pairwise scatterplots of log_{10} transformed counts overlapping all CLIP-seq peaks identified by PIPE-CLIP (including those not enriched in 3xFLAG-CsrA over untagged strains) and Spearman correlation coefficients for each biological replicate of a. wild type untagged CLIP-seq samples and b. 3xFLAG-CsrA CLIP-seq samples. c. An MA plot for all CLIP-seq peaks identified by PIPE-CLIP showing M (log_2 fold change of 3xFLAG-CsrA over untagged controls) and A (average normalized counts of RNA overlapping these peaks from the paired RNA libraries from the ribosome profiling analysis) with significantly enriched CLIP-seq peaks in black. This analysis suggests that identification of a CLIP-seq peak does not strongly depend on the level of expression of the associated RNA transcript.

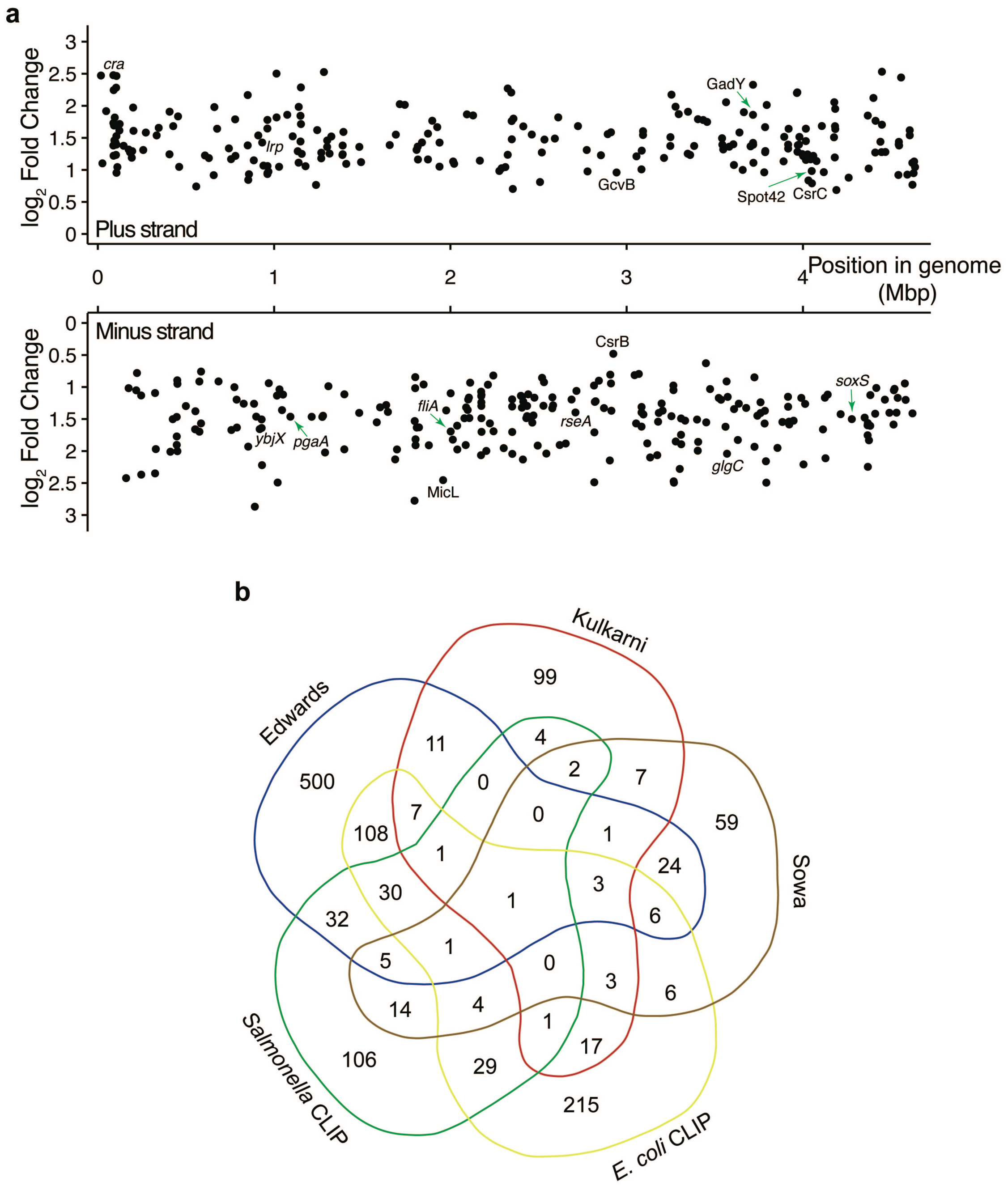
a
log2 Fold Change
Plus strand
cra
lrp
GcvB
GadY
Spot42
CsrC
Position in genome (Mbp)
Minus strand
CsrB
ybjX
pgaA
fliA
rseA
MicL
glgC
soxS
b
Kulkarni
Edwards
Sowa
Salmonella CLIP
E. coli CLIP
99
11
4
500
0
2
7
7
0
108
1
59
1
24
30
3
1
32
6
5
1
0
3
6
14
4
1
17
106
29
215

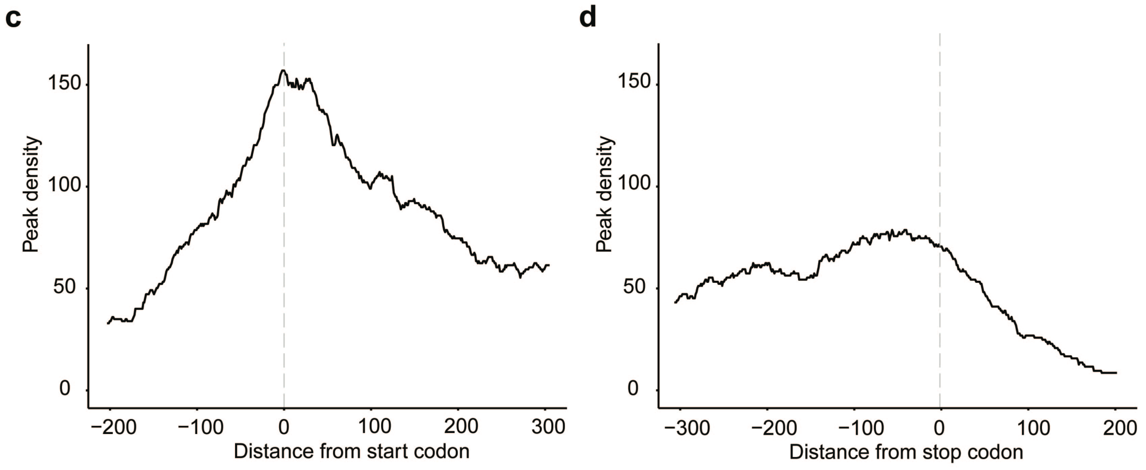

Supplementary Figure 3: CLIP-seq identified novel and known direct CsrA targets

a. Position of significant CsrA CLIP-seq peaks in the genome and their $\log_2$ transformed fold enrichment over the untagged control samples. Selected well-characterized CsrA binding partners and those discussed further in this work are labeled. b. Overlap of CLIP-seq results from this study with previous studies that predicted CsrA targets in *E. coli* based on a systematic evolution of ligands by exponential enrichment (SELEX) derived consensus motif (Kulkarni et al.[1]), overexpressed CsrA and sequenced copurifying RNA (Edwards et al.[2]), and identified targets by looking for changes in gene expression in RNAs that also formaldehyde crosslinked with CsrA *in vivo* (Sowa et al.[3]). *E. coli* homologs of genes identified in a recently published CLIP-seq analysis in *Salmonella enterica* serovar Typhimurium strain SL1344 are also shown (Holmqvist et al.[4]) c. Position of peaks that overlap 5'-untranslated regions (5'-UTRs) and coding sequences relative to the associated start codon. d. Position of peaks that overlap 3'-UTRs and coding sequences relative to the associated stop codon.

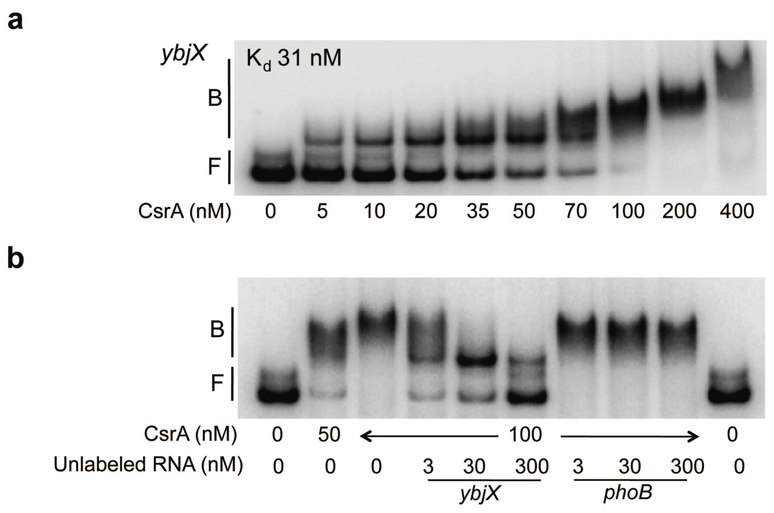

Supplementary Figure 4: CsrA binds to the 3’ untranslated region (UTR) of *ybjX*

a. Electrophoretic mobility shift assay (EMSA) showing interaction of CsrA with the 3’-UTR of *ybjX* (+1 to +199 relative to the stop codon). F, free RNA; B, bound RNA. K_d, apparent dissociation constant. b. Competition assay establishing CsrA-*ybjX* RNA binding specificity. Unlabeled *ybjX* was an effective competitor, whereas the non-specific competitor *phoB* RNA was not.

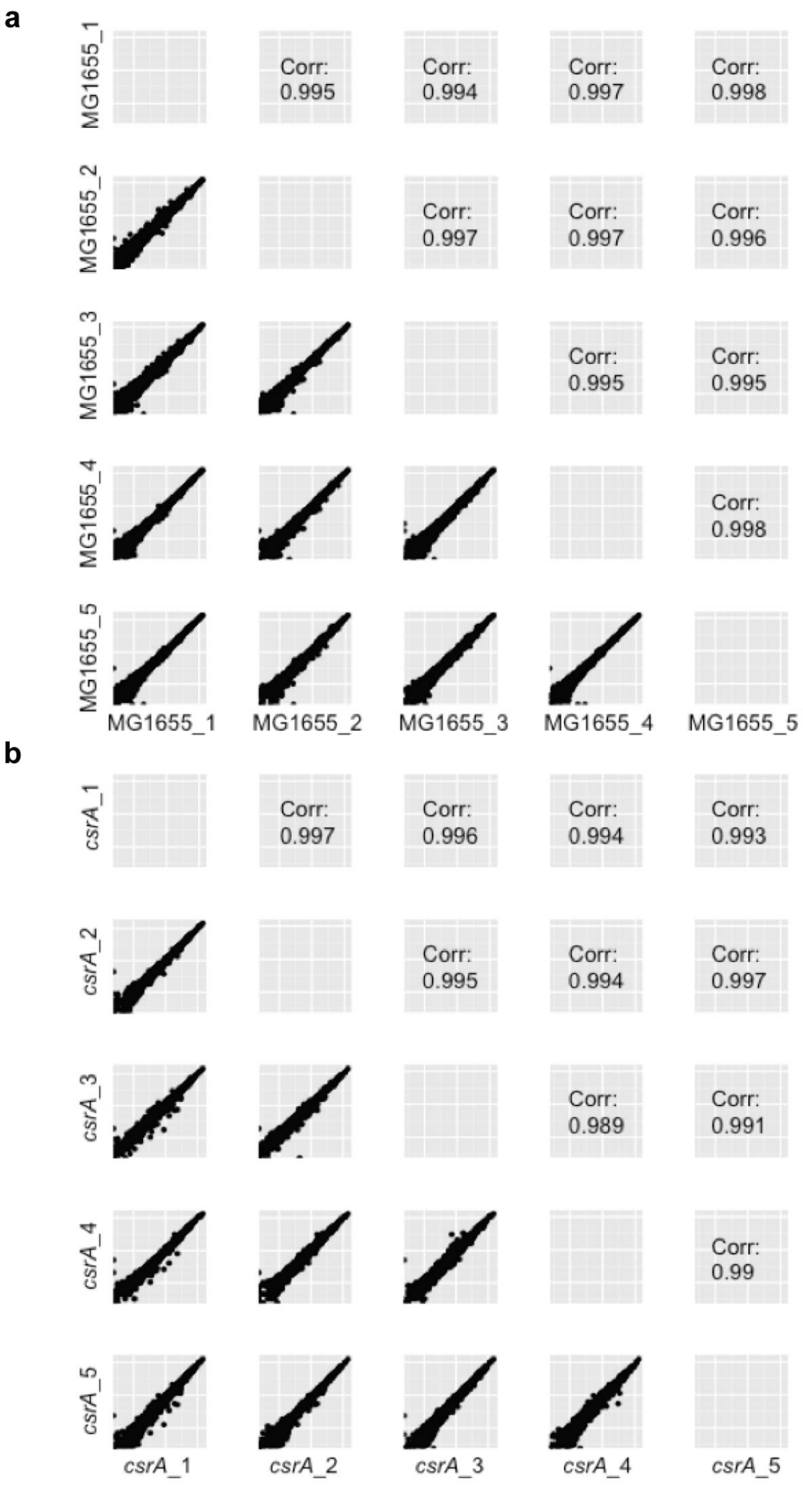
a
MG1655_1
MG1655_2
MG1655_3
MG1655_4
MG1655_5
Corr: 0.995
Corr: 0.994
Corr: 0.997
Corr: 0.998
Corr: 0.997
Corr: 0.997
Corr: 0.996
Corr: 0.995
Corr: 0.995
Corr: 0.998
b
csrA_1
csrA_2
csrA_3
csrA_4
csrA_5
Corr: 0.997
Corr: 0.996
Corr: 0.994
Corr: 0.993
Corr: 0.995
Corr: 0.994
Corr: 0.997
Corr: 0.989
Corr: 0.991
Corr: 0.99

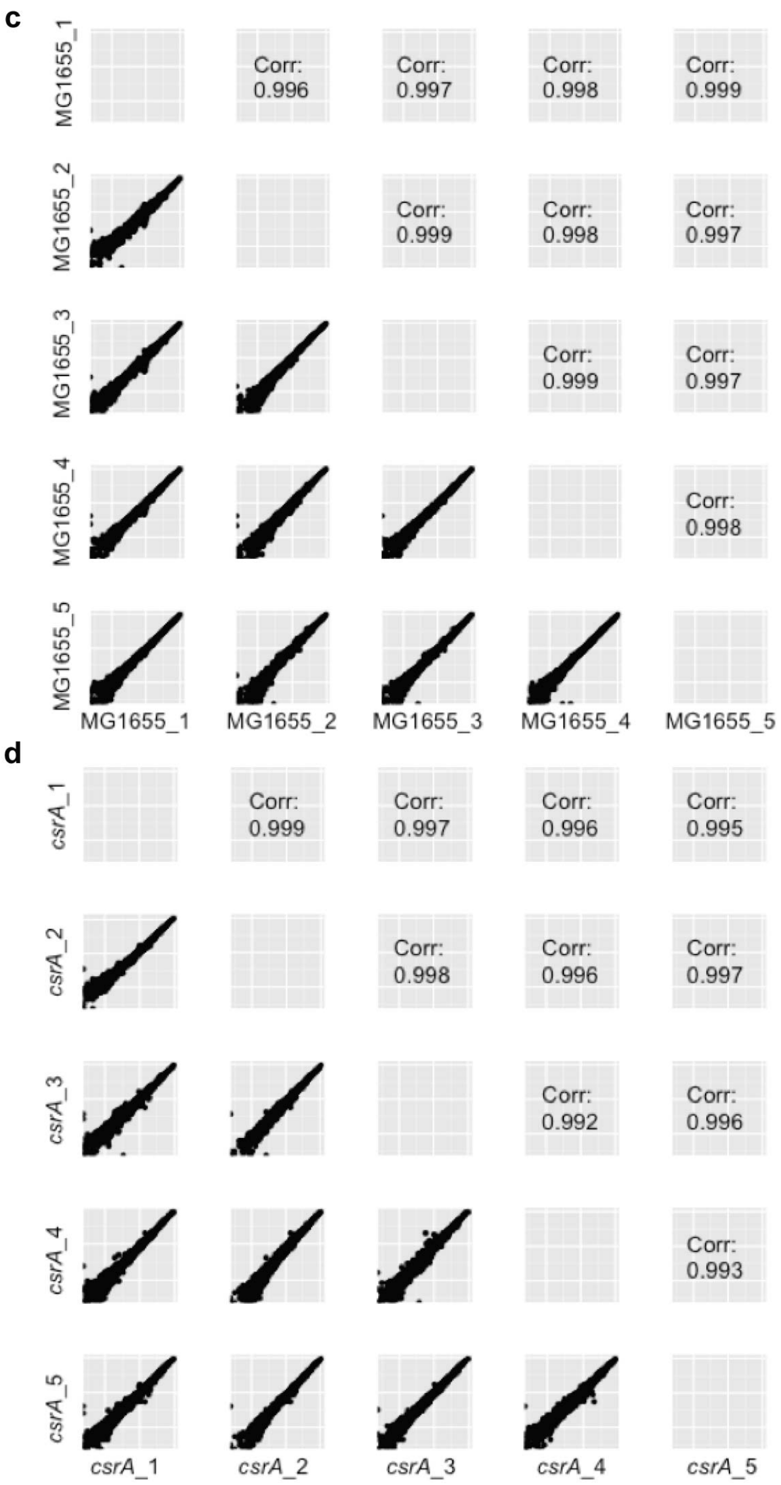

Supplementary Figure 5: Ribosome profiling analysis is reproducible
Pairwise scatterplots of $\log_{10}$ transformed counts per million and Spearman correlation coefficients for each biological replicate of a. Wild type ribosome protected fragment (RPF) samples. b. *csrA* mutant RPF samples. c. Wild type RNA samples. d. *csrA* mutant RNA samples.

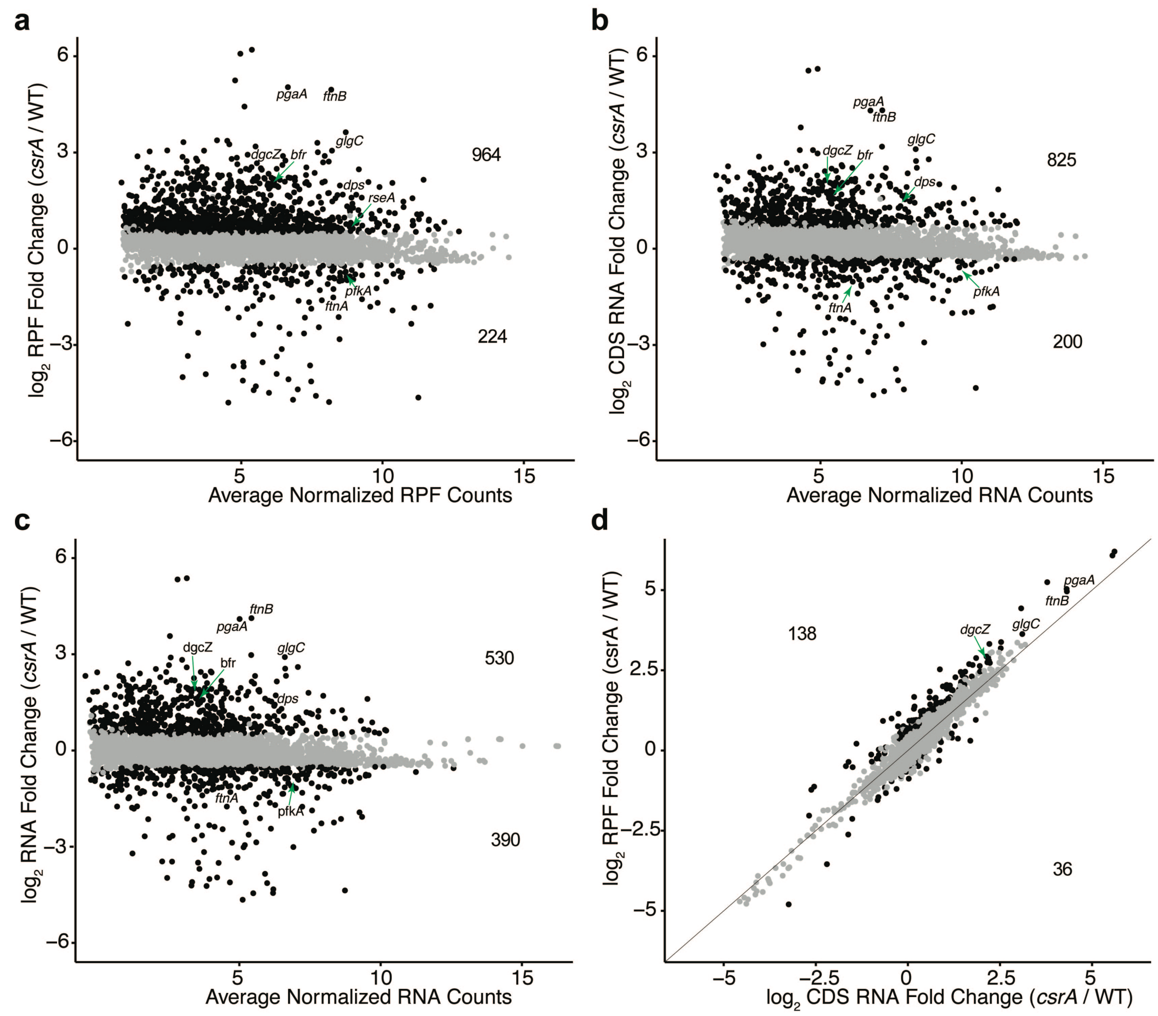

Supplementary Figure 6: CsrA has a vast impact on both translation and RNA abundance

a-c. Number of differentially expressed genes and MA plots showing M ($\log_2$ fold change) and A (average normalized counts) between the wild type and *csrA* mutant strains with significant changes in black. a. Changes in RPF abundance. b. Changes in RNA abundance of protein coding genes. c. Changes in RNA abundance of all genes. d. Log_2 transformed fold changes in translation (RPF abundance) versus RNA abundance where genes with significant changes in translation efficiency are shown in black and the exact numbers shown. Known CsrA targets and genes discussed further in the main text are labeled.

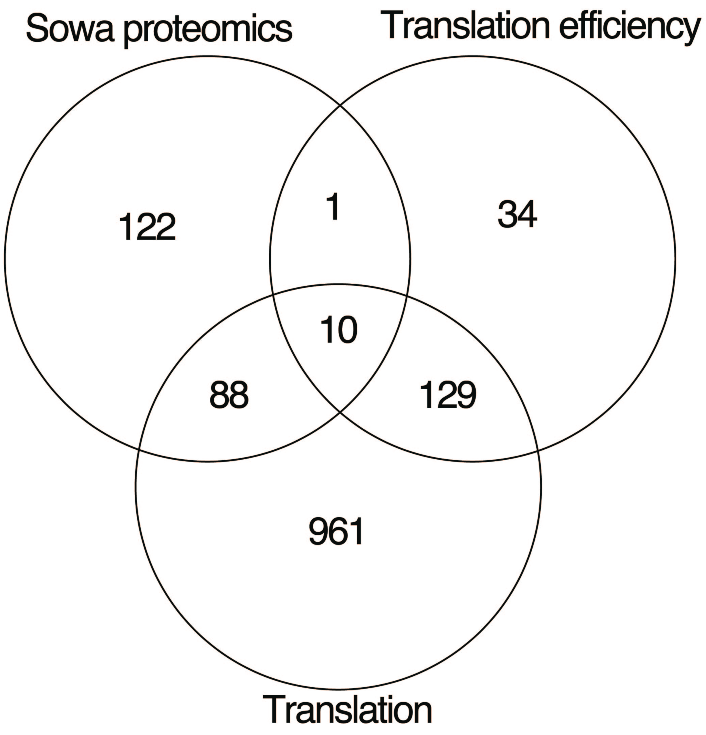

Supplementary Figure 7: Overlap of our ribosome profiling data with proteomics data from Sowa et al.[3].
Sowa et al. analyzed the effect of CsrA on protein levels at one time point in M9 minimal medium plus 0.2% glucose before imposing carbon starvation. The Venn diagram shows the overlap between the genes significantly different between the wild type and *csrA* mutant in translation and/or translation efficiency in our study and the proteomics results of in Sowa et al.

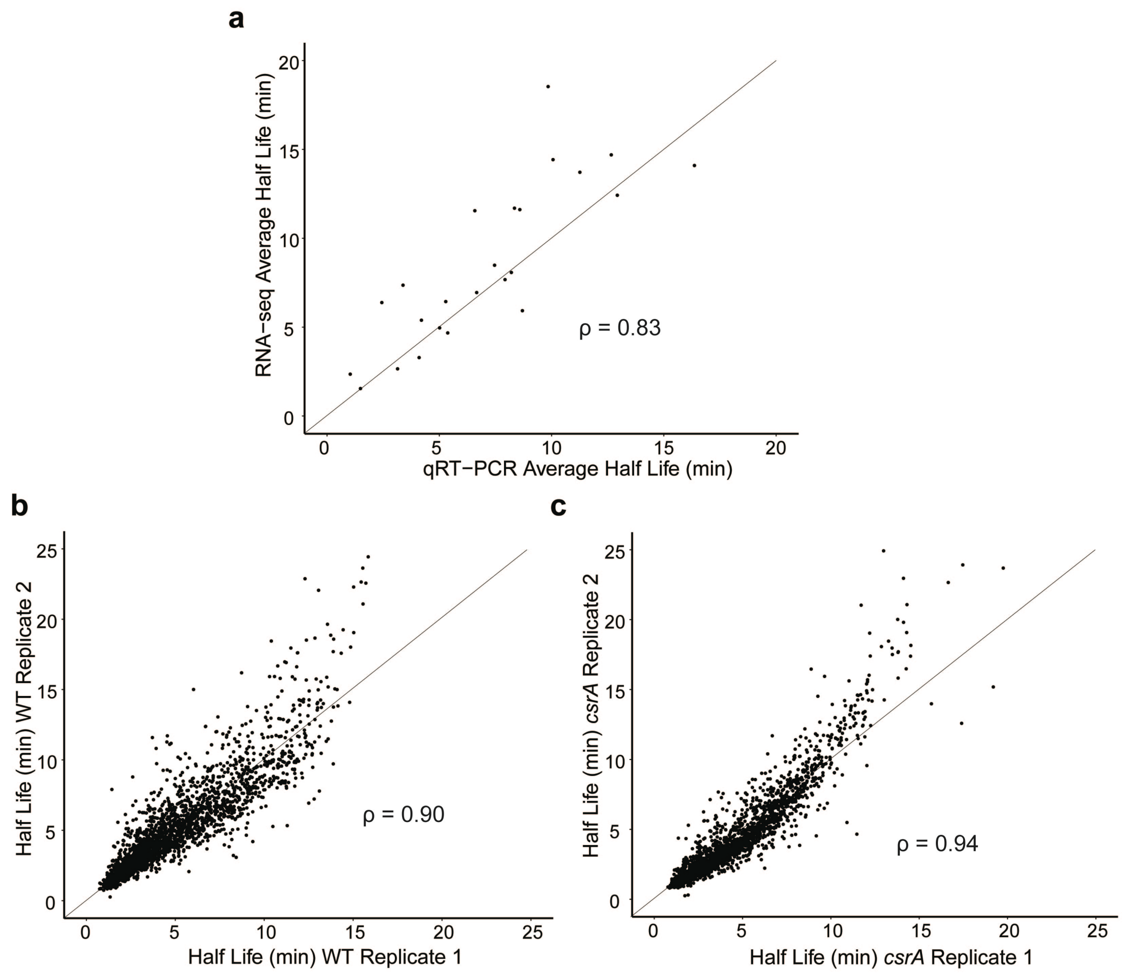

Supplementary Figure 8: RNA-seq analysis of RNA stability is reliable and reproducible

a. Scatterplot of RNA half-lives calculated with RNA-seq analysis and qRT-PCR and Spearman correlation coefficient. b, c. Scatterplot of RNA half-lives calculated from each replicate of RNA-seq analysis with associated Spearman correlation coefficients: Wild type (b) and *csrA* mutant (c) strains.

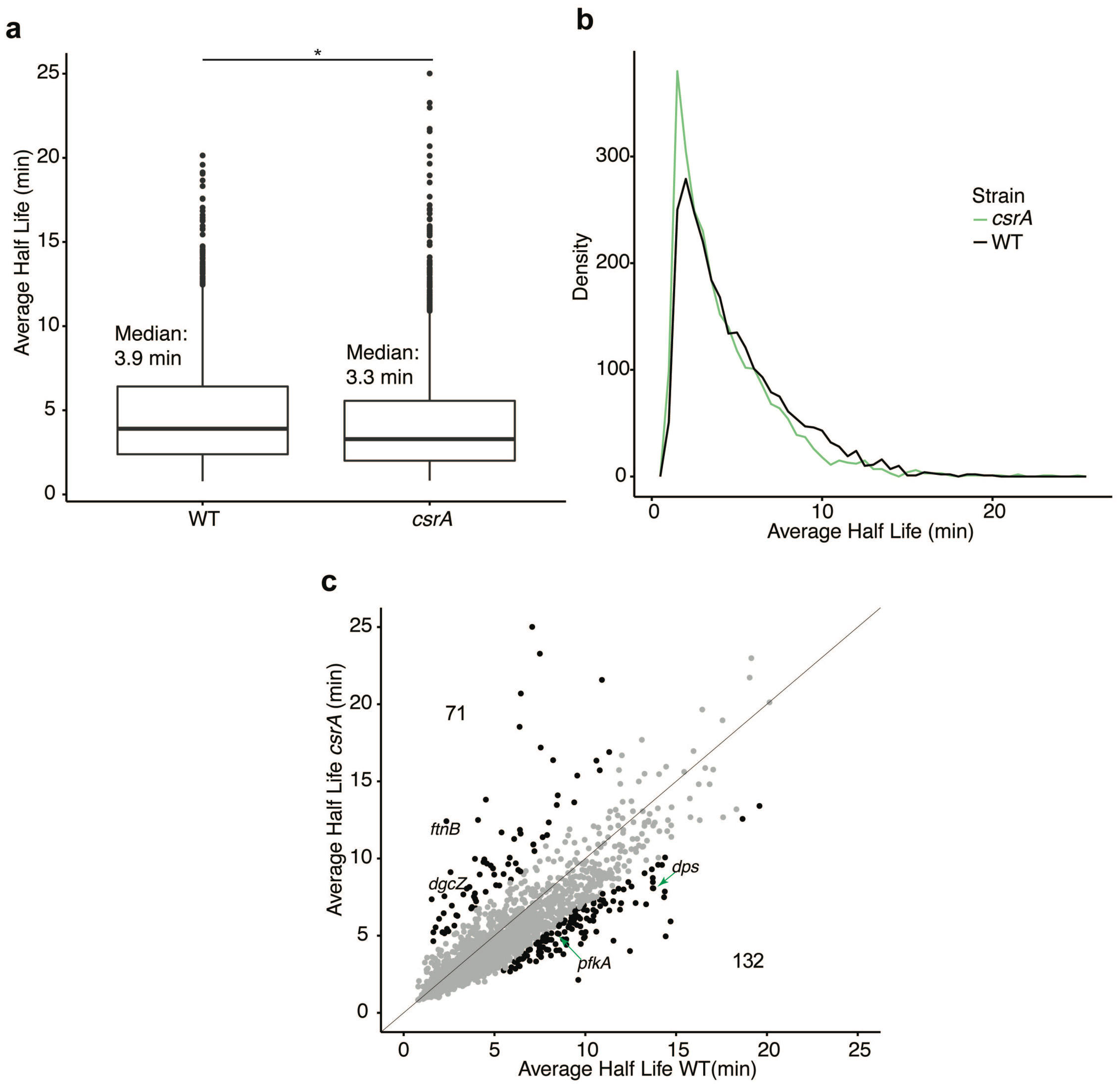

Supplementary Figure 9: CsrA has a global effect on RNA stability
a. Boxplot of RNA half-lives calculated with RNA-seq analysis with associated median. The distributions are significantly different when analyzed with the Wilcox rank sum test ($p = 2.03x10^{-15}$). b. Density plot of average RNA half-lives for the wild type (black) and *csrA* mutant (green) strains. c. Average half-lives of RNAs in wild type and *csrA* mutant strains where genes with significant changes in their half lives are shown in black and the exact numbers shown. Known CsrA targets and genes discussed further in the main text are labeled.

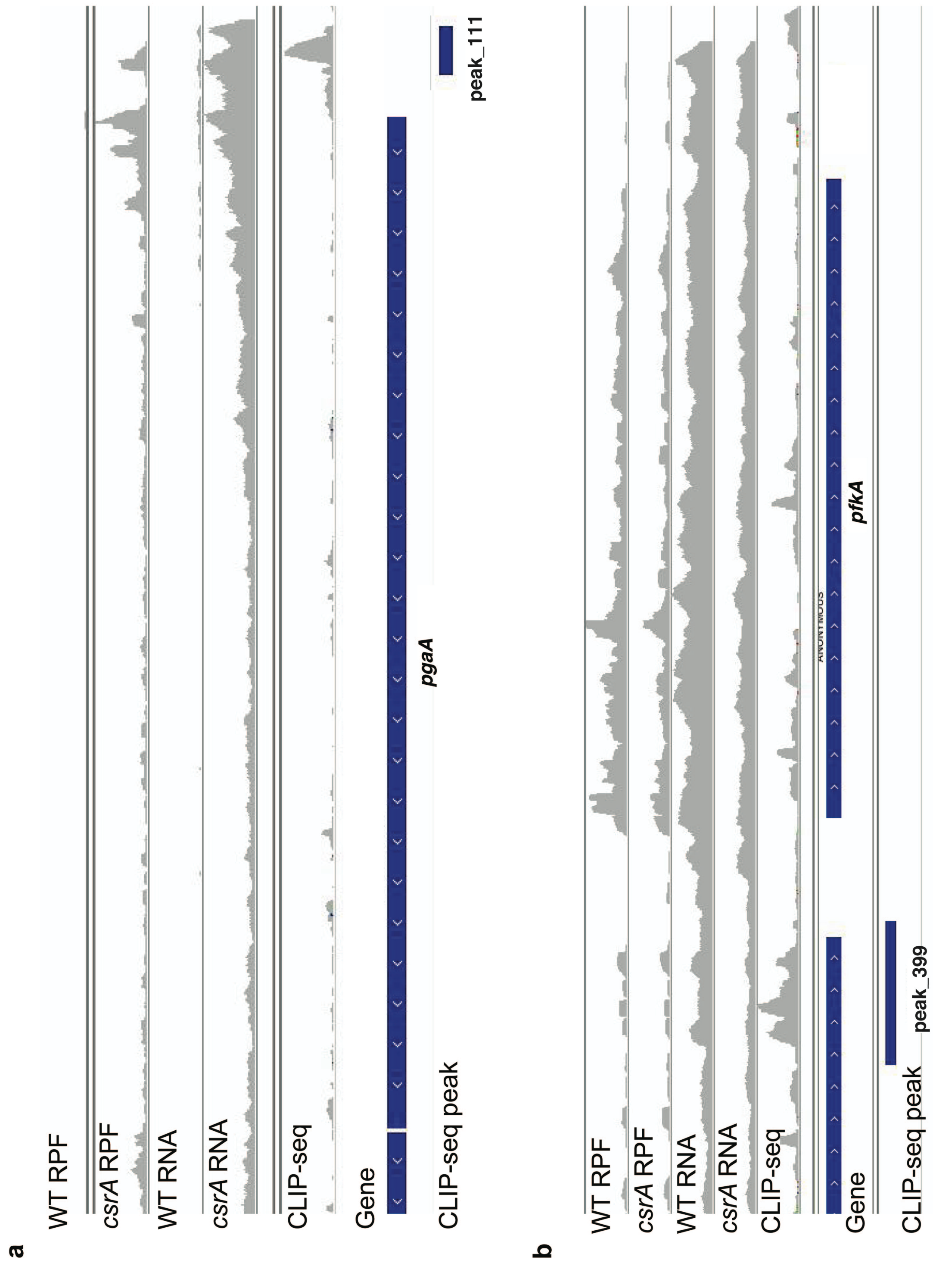
a
WT RPF
csrA RPF
WT RNA
csrA RNA
CLIP-seq
Gene
CLIP-seq peak
pgaA
peak_111
b
WT RPF
csrA RPF
WT RNA
csrA RNA
CLIP-seq
Gene
CLIP-seq peak
pfkA
peak_399

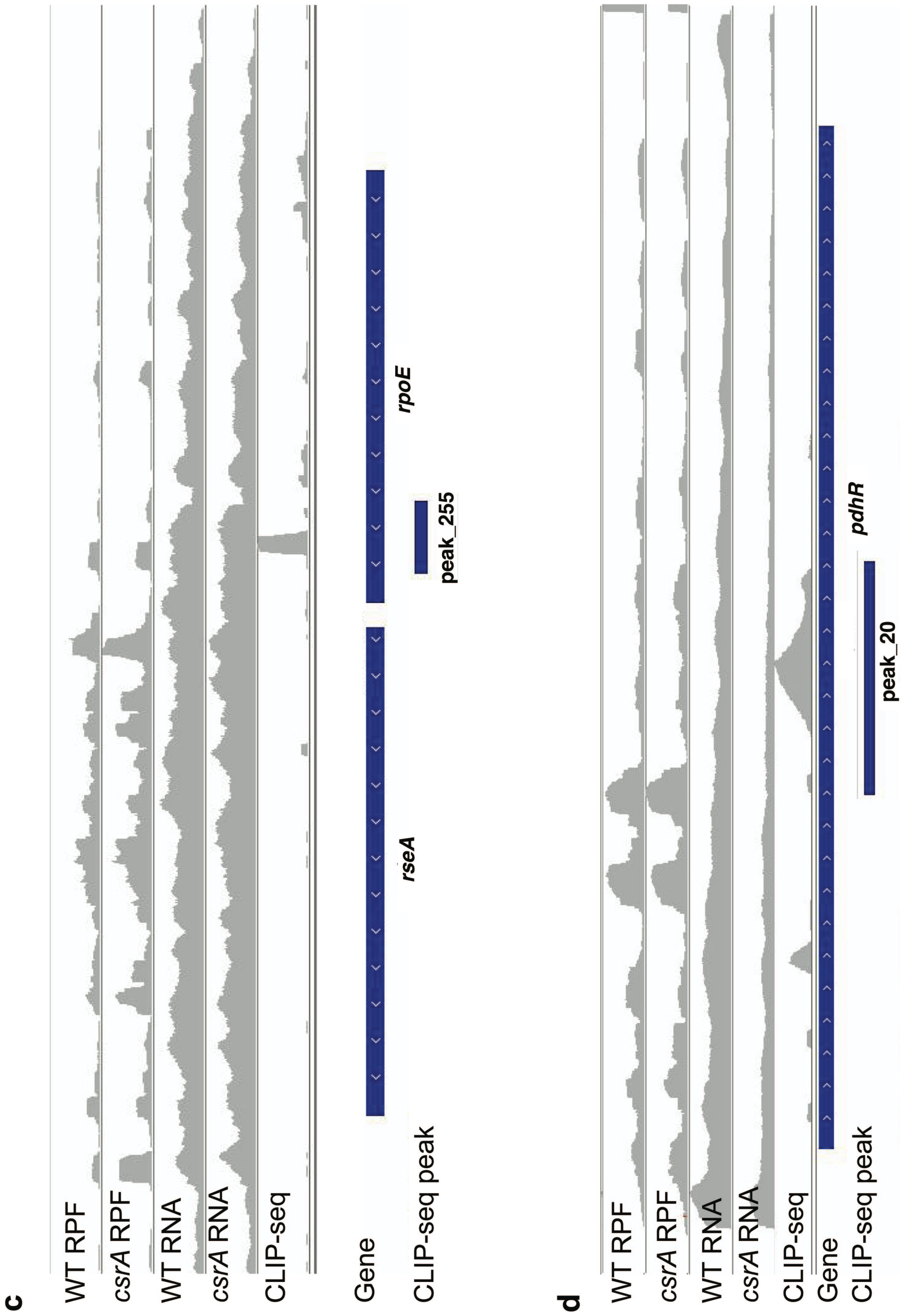
c
WT RPF
csrA RPF
WT RNA
csrA RNA
CLIP-seq
Gene
CLIP-seq peak
rseA
rpoE
peak_255
d
WT RPF
csrA RPF
WT RNA
csrA RNA
CLIP-seq
Gene
CLIP-seq peak
pdhR
peak_20

e

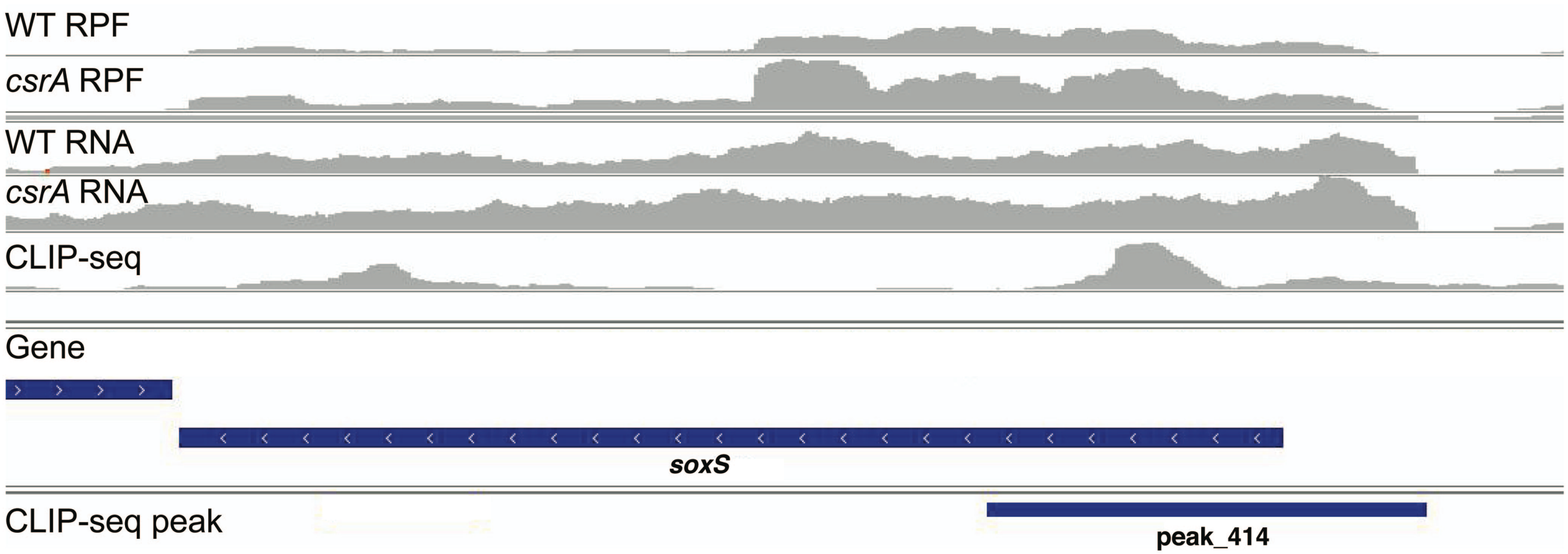

Supplementary Figure 10: Genome browser views of ribosome profiling, RNA-seq, and CLIP-seq reads for genes associated with CLIP-seq peaks and different regulatory effects. Ribosome protected fragments (RPF) and RNA data for wild type and *csrA* mutant samples were normalized and shown in the same scale for each sample type. A single representative biological replicate is shown. The CLIP-seq reads shown are a combination of the 3xFLAG-CsrA replicates. a. The CsrA CLIP-seq peak overlapped a site in the *pgaA* mRNA involved in stimulating Rho-dependent transcription termination[6]. CsrA also binds to sites that lead to direct repression of *pgaA* translation initiation[5]. Our data showed that CsrA represses *pgaA* at the level of RNA abundance, translation, and translation efficiency. b. The CLIP-seq peak overlapped the 5-UTR of *pfkA*[7]. Our data showed that CsrA stabilized the *pfkA* transcript, which is also reflected in activation of its RNA abundance and translation. c. The CsrA CLIP-seq peak overlapped the 3' end of the *rpoE* coding sequence and 5'-UTR of *rseA*[7]. CsrA repressed the translation of *rseA*. d. The CsrA CLIP-seq peaks was deep within the *pdhR* coding sequence, and CsrA repressed its RNA abundance. e. The CsrA CLIP-seq peak overlapped the *soxS* 5-UTR and coding sequence. CsrA repressed the translation and RNA of *soxS*.

a

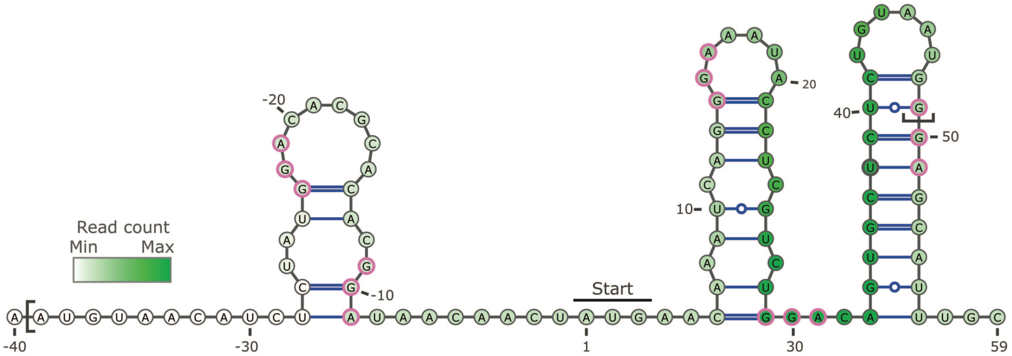

b

Gene	RPF	CDS.RNA	TE	RNA	Stability
cstA	1	0	0	0	0

Supplementary Figure 11: Despite apparent lack of post-transcriptional regulation of *cstA*, the combination of CLIP-seq and transcriptomics data supports *cstA* as a direct CsrA target as previously published
a. Previous work demonstrated that CsrA binds to the *cstA* transcript at four sites, one of which overlaps its Shine-Dalgarno sequence (-11 to -9 relative to the start codon)[8]. Bound CsrA represses translation initiation of *cstA* by blocking ribosome binding. In this study, we identified a CLIP-seq peak (between the brackets) overlapping the region where CsrA binds *in vitro*, and the CLIP-seq reads from all 5 replicates are overlaid in green. GGA motifs are outlined in pink. b. Our transcriptomics data showed that CsrA repressed *cstA* translation but not its translation efficiency. Nevertheless, a CLIP-seq peak overlapping *cstA* and a change in translation was strong evidence for direct regulation that is mirrored in published work[8].

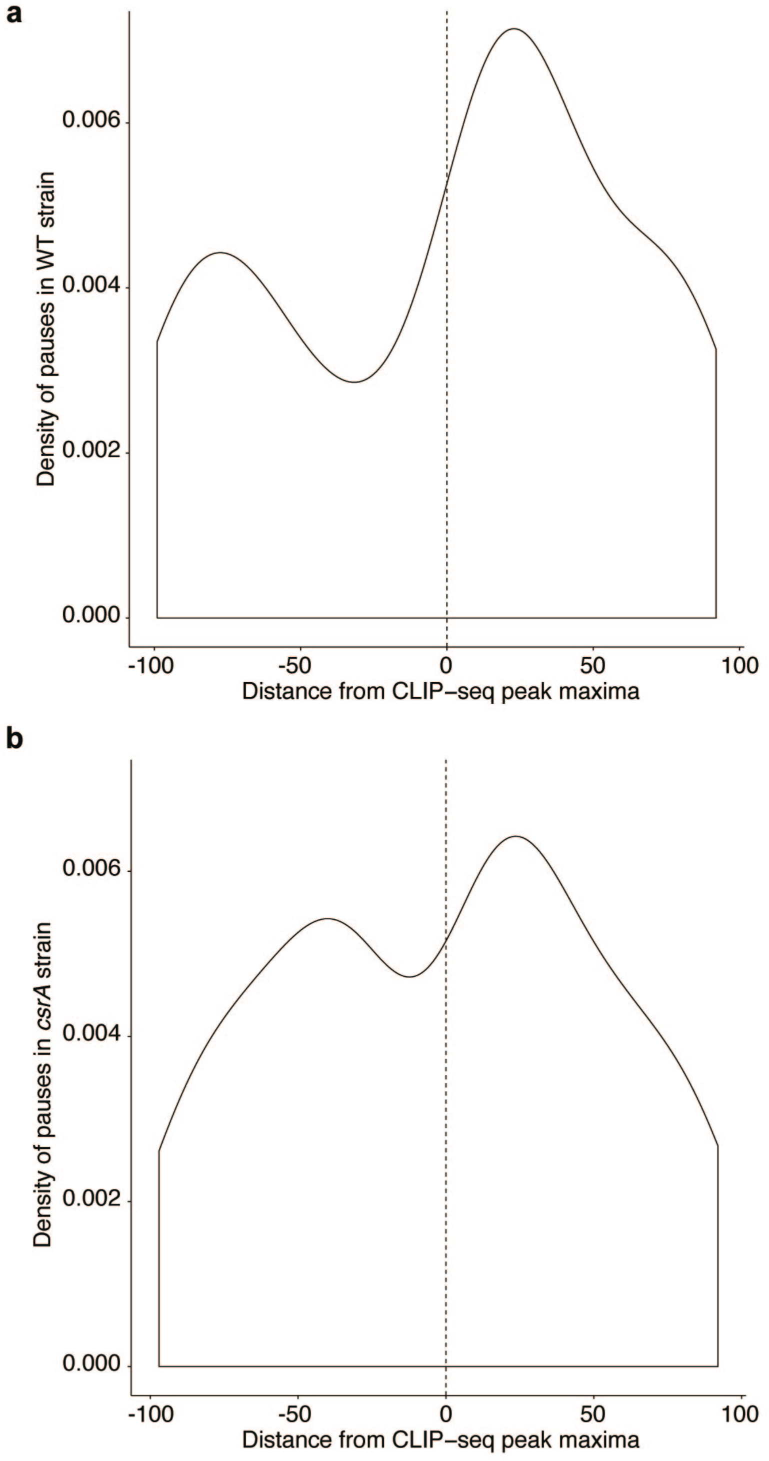
a
Density of pauses in WT strain
0.000
0.002
0.004
0.006
-100
-50
0
50
100
Distance from CLIP−seq peak maxima
b
Density of pauses in csrA strain
0.000
0.002
0.004
0.006
-100
-50
0
50
100
Distance from CLIP−seq peak maxima

c

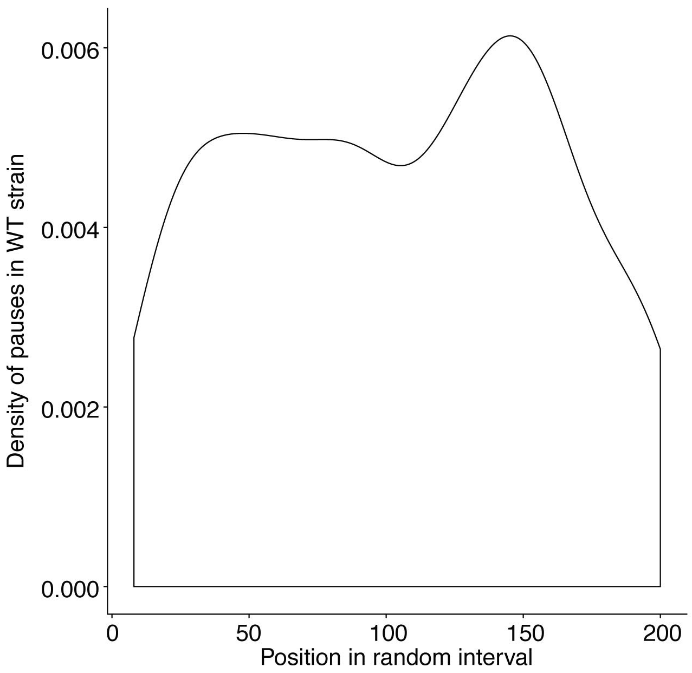
0.006
0.004
0.002
0.000
Density of pauses in WT strain
0
50
100
150
200
Position in random interval

d

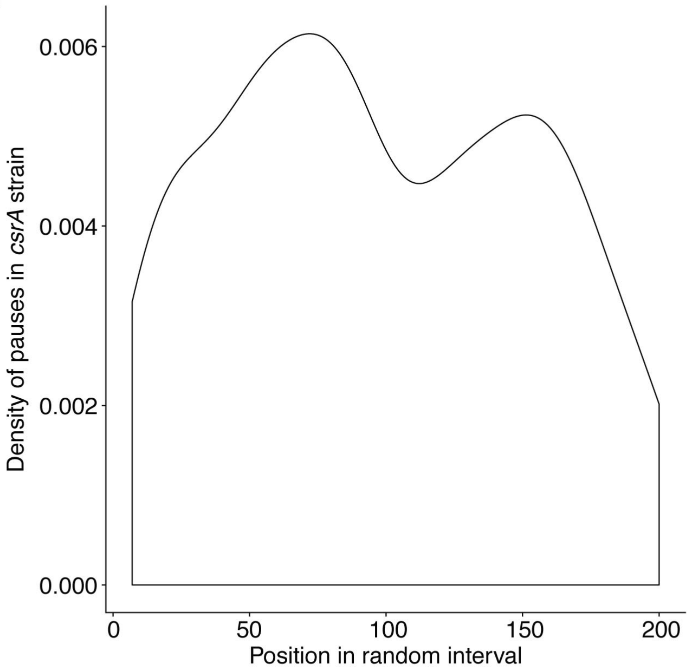
0.006
0.004
0.002
0.000
Density of pauses in *csrA* strain
0
50
100
150
200
Position in random interval

e

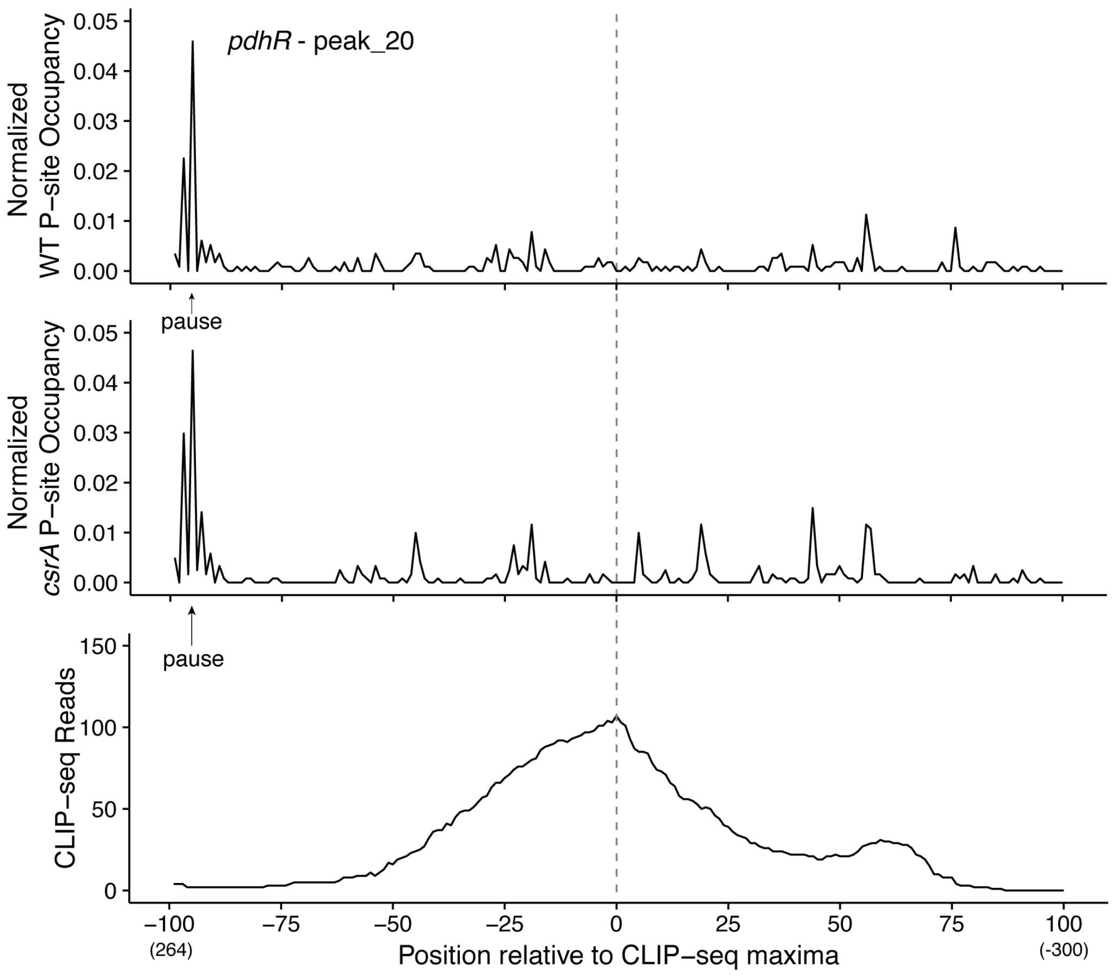
pdhR - peak_20
Normalized
WT P−site Occupancy
0.05
0.04
0.03
0.02
0.01
0.00
pause
Normalized
csrA P−site Occupancy
0.05
0.04
0.03
0.02
0.01
0.00
pause
CLIP−seq Reads
150
100
50
0
−100
(264)
−75
−50
−25
0
25
50
75
100
(-300)
Position relative to CLIP−seq maxima

f

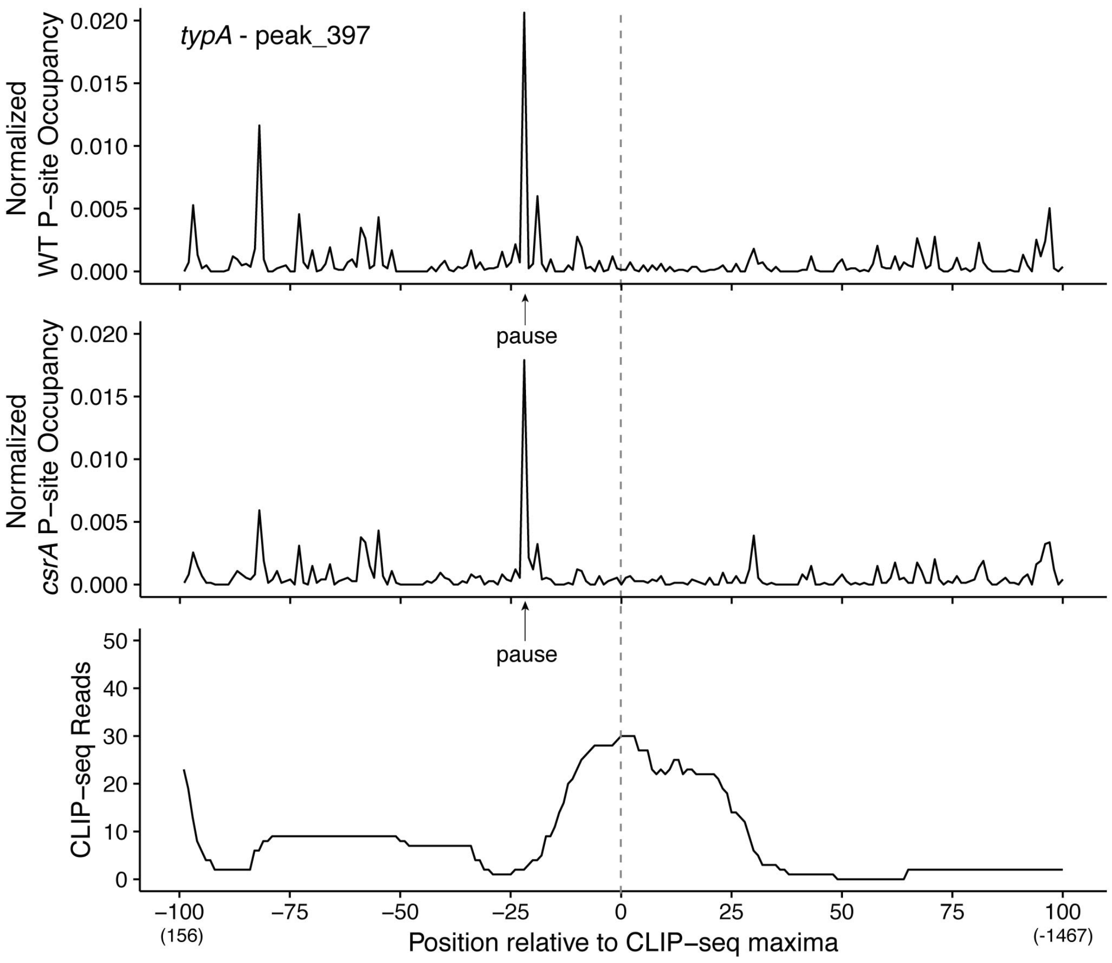
typA - peak_397
Normalized
WT P–site Occupancy
0.020
0.015
0.010
0.005
0.000
pause
Normalized
csrA P–site Occupancy
pause
CLIP–seq Reads
50
40
30
20
10
0
−100
(156)
−75
−50
−25
0
25
50
75
100
(-1467)
Position relative to CLIP–seq maxima

g

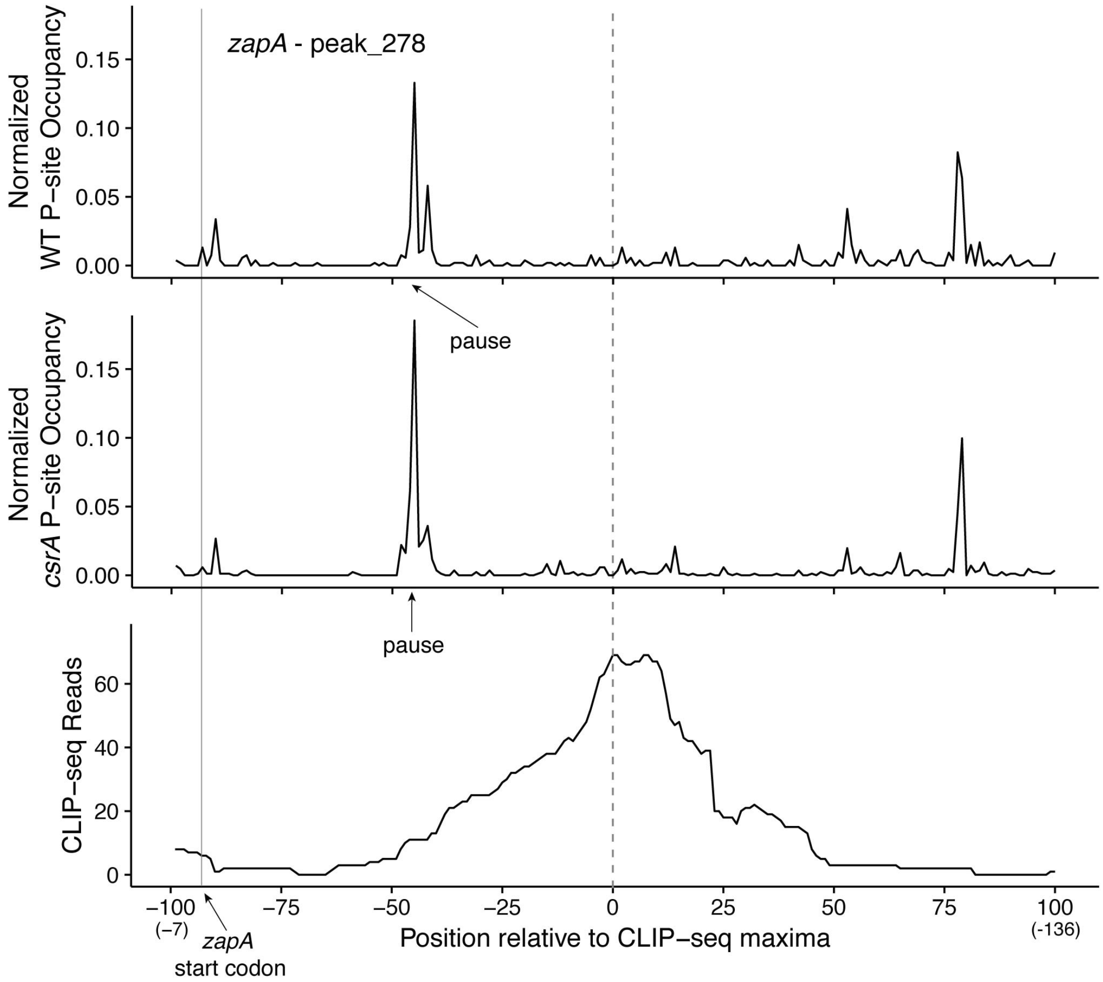
zapA - peak_278
Normalized
WT P-site Occupancy
0.15
0.10
0.05
0.00
pause
Normalized
csrA P-site Occupancy
0.15
0.10
0.05
0.00
pause
CLIP-seq Reads
60
40
20
0
−100
(−7)
−75
−50
−25
0
25
50
75
100
(-136)
zapA
start codon
Position relative to CLIP-seq maxima

h

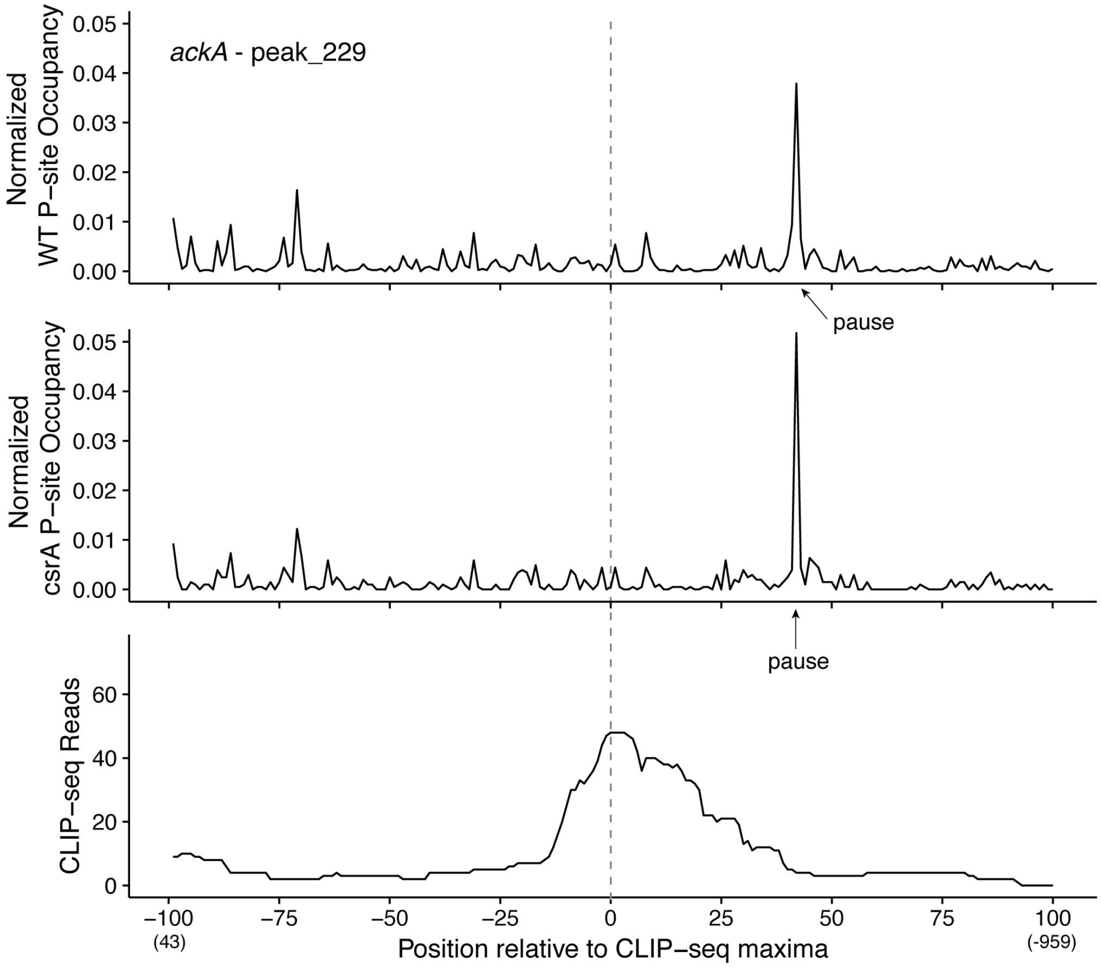
ackA - peak_229
Normalized
WT P−site Occupancy
0.05
0.04
0.03
0.02
0.01
0.00
pause
Normalized
csrA P−site Occupancy
0.05
0.04
0.03
0.02
0.01
0.00
pause
CLIP−seq Reads
60
40
20
0
−100
(43)
−75
−50
−25
0
25
50
75
100
(-959)
Position relative to CLIP−seq maxima

i

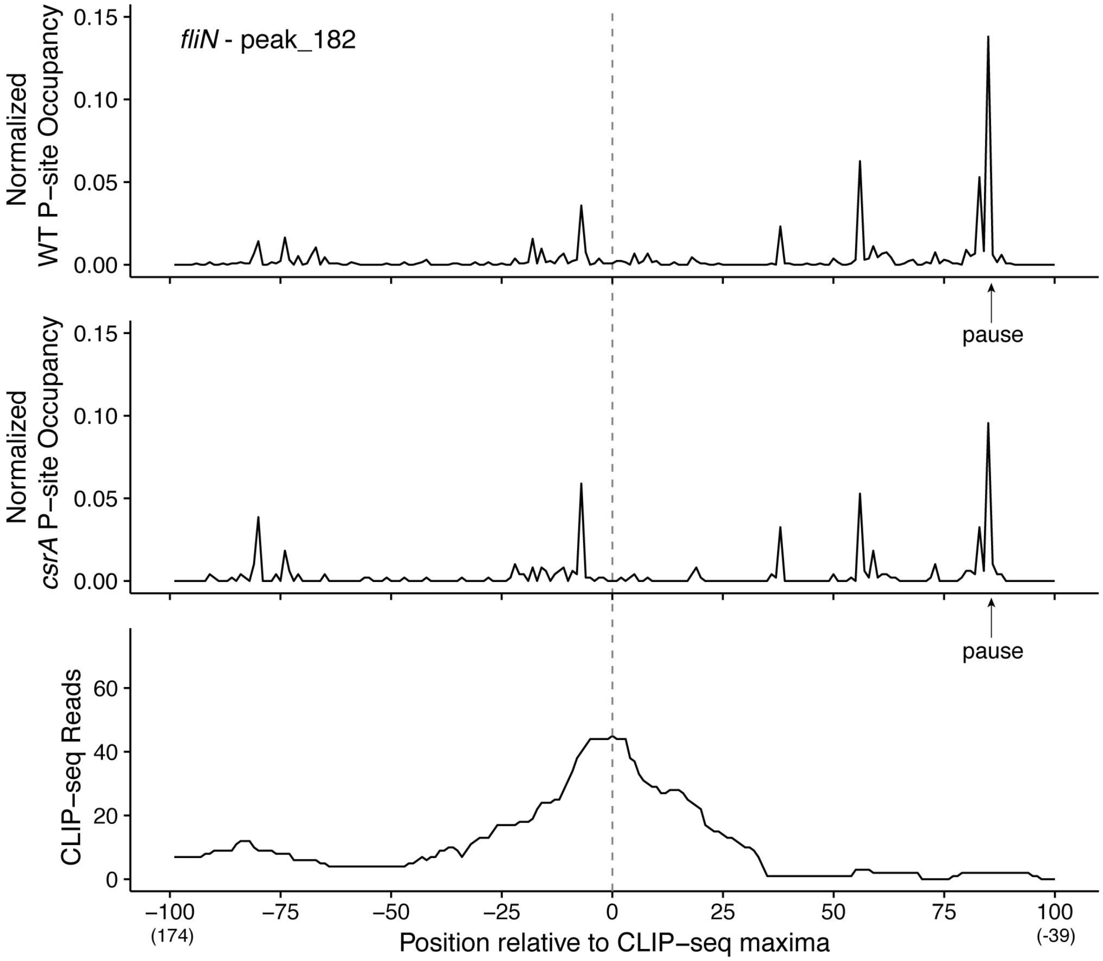

fliN - peak_182
Normalized
WT P–site Occupancy
0.15
0.10
0.05
0.00
pause
Normalized
csrA P–site Occupancy
0.15
0.10
0.05
0.00
pause
CLIP–seq Reads
60
40
20
0
−100
(174)
−75
−50
−25
0
25
50
75
100
(-39)
Position relative to CLIP–seq maxima

j

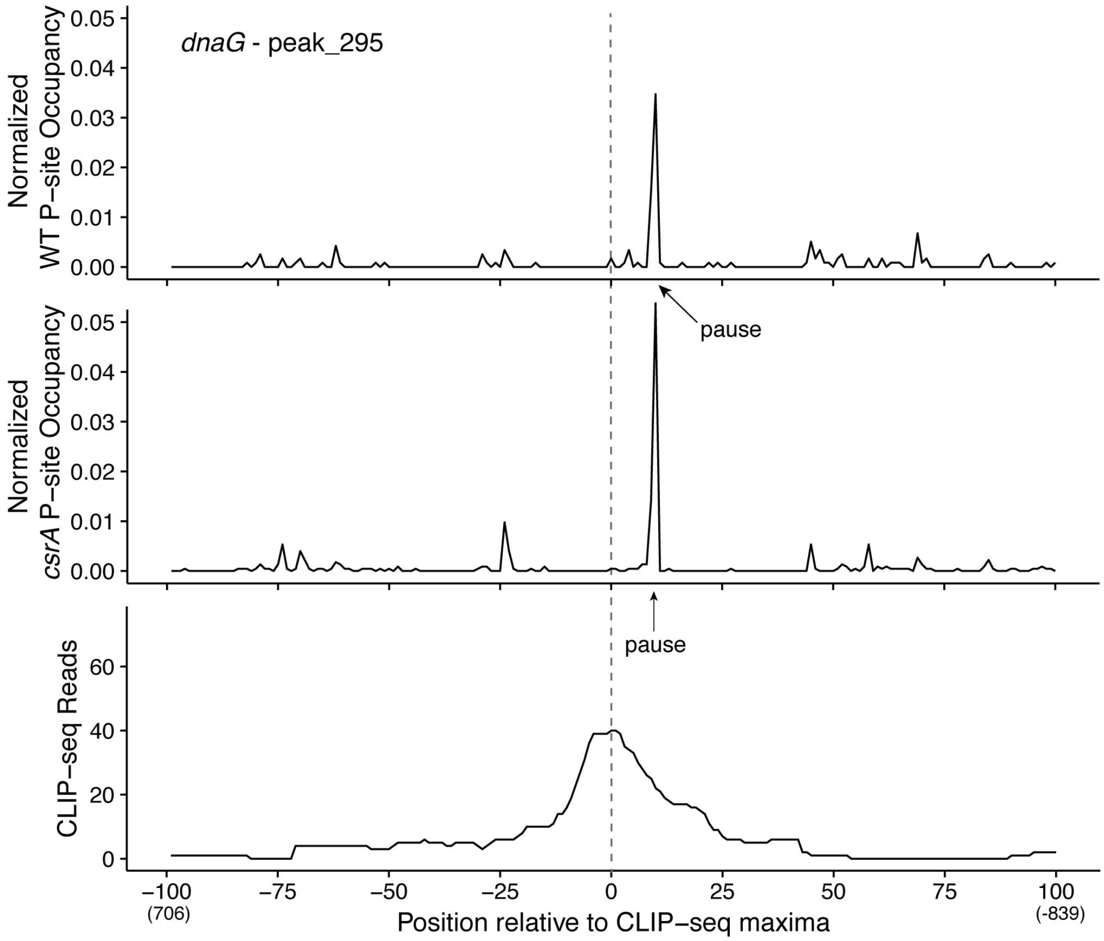

Supplementary Figure 12: Some CLIP-seq peaks are associated with ribosome pauses.
Plots are centered at the CLIP-seq maxima, or position with the largest number of read counts within the peak. Ribosome occupancy was assigned at the P-site and normalized to the total occupancy of trimmed coding sequence, and pauses are defined as normalized occupancy greater than 10 fold over the average for each trimmed coding sequence (see Materials and Methods). a-b. Kernel density estimate of pauses within 100 bases flanking the maxima of the 39 CLIP-seq peaks associated with ribosome pauses (as defined in the Materials and Methods). c-d. Kernel density estimate of pauses across random 200 base intervals of CDS of sufficient expression for analysis of ribosome pausing. e-j. Selected CLIP-seq peaks associated with ribosome pauses. Distances in parentheses are relative to the gene start and stop codons. Occupancy data is shown from a single representative biological replicate for WT and *csrA* mutant strains. The CLIP-seq reads shown are a combination of the 3xFLAG-CsrA replicates.

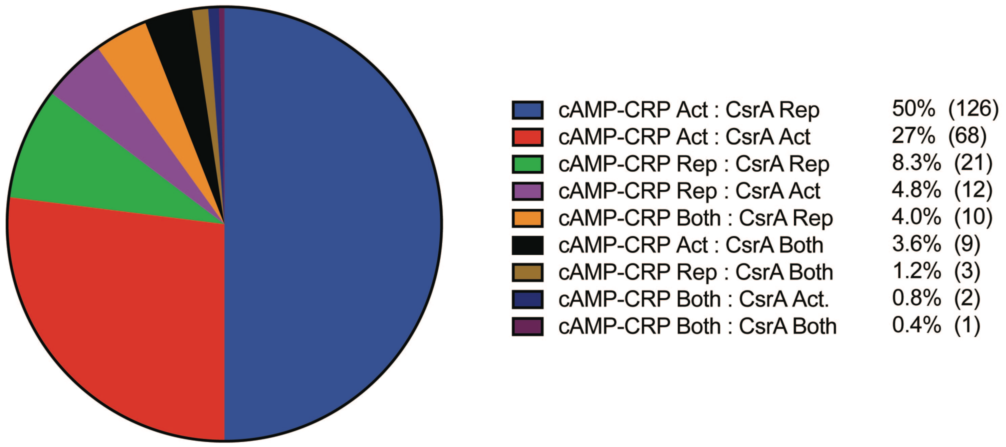

Supplementary Figure 13: Overlap between cAMP-CRP and CsrA regulons
The cAMP-CRP regulon (547 genes) was extracted from RegulonDB[9] and compared to genes differentially expressed between wild type and *csrA* mutant strains in any of the expression analyses. The pie chart depicts the overlap in these lists with respect to the direction of the effects (Rep: repressed by, Act: activated by, Both: both activated and repressed by), percent of the total overlap, and exact number of genes in each category.

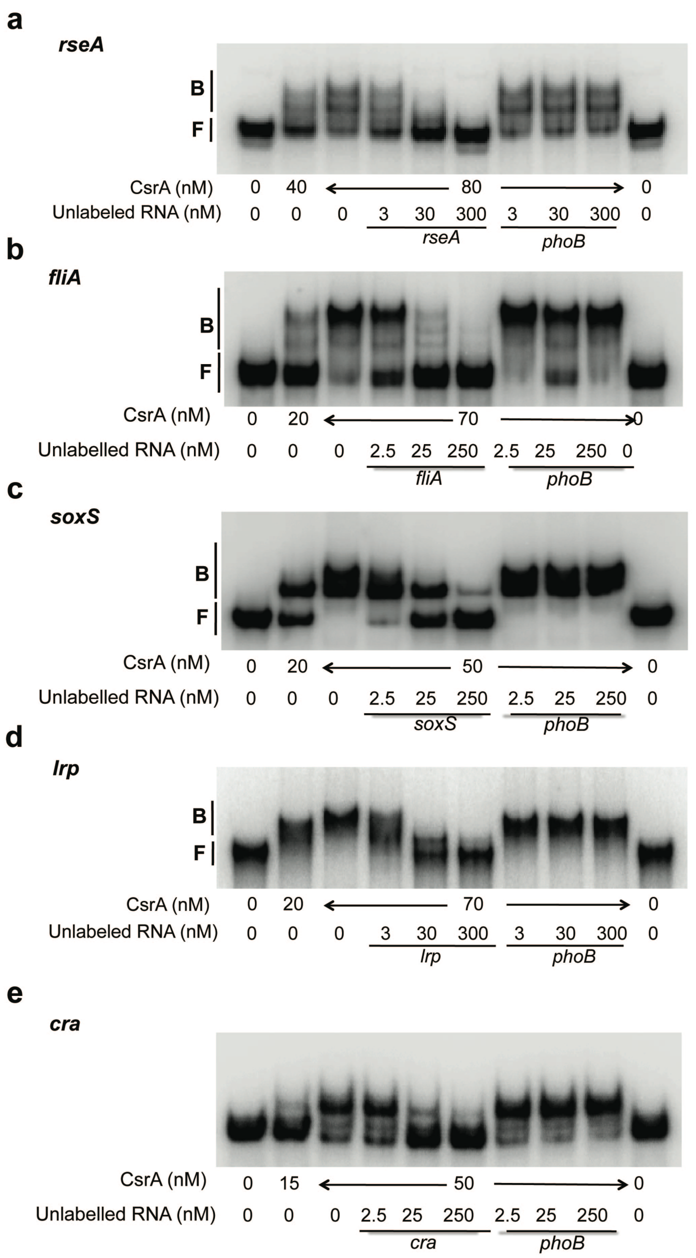
a
rseA
B
F
CsrA (nM) 0 40 80 0
Unlabeled RNA (nM) 0 0 0 3 30 300 3 30 300 0
rseA
phoB
b
fliA
B
F
CsrA (nM) 0 20 70 0
Unlabelled RNA (nM) 0 0 0 2.5 25 250 2.5 25 250 0
fliA
phoB
c
soxS
B
F
CsrA (nM) 0 20 50 0
Unlabelled RNA (nM) 0 0 0 2.5 25 250 2.5 25 250 0
soxS
phoB
d
lrp
B
F
CsrA (nM) 0 20 70 0
Unlabeled RNA (nM) 0 0 0 3 30 300 3 30 300 0
lrp
phoB
e
cra
CsrA (nM) 0 15 50 0
Unlabelled RNA (nM) 0 0 0 2.5 25 250 2.5 25 250 0
cra
phoB

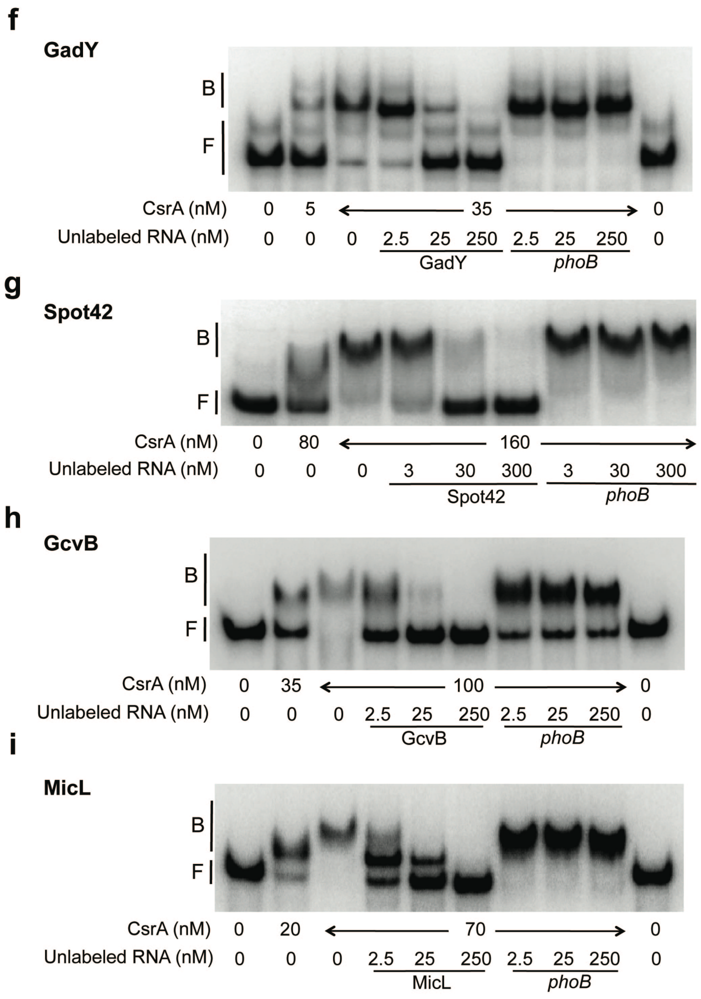

Supplementary Figure 14: The *in vitro* binding interactions of CsrA are specific

Competition electrophoretic mobility shift assays (EMSA) showing specificity of interactions of CsrA with each of the indicated transcripts. F, free RNA; B, bound RNA. Whereas unlabeled specific RNAs were effective competitors as indicated, the non-specific competitor *phoB* RNA was not.

a

TA system	Type	Peak upstream of	Expression
YafQ/DinJ	II	Toxin	- RPF *yafQ*
RelE/RelB	II	Toxin	N/A
YefM/YoeB	II	Toxin	N/A
MazE/MazF	II	Toxin	N/A
PrlF/YhaV	II	Toxin	N/A
ChpS/ChpB	II	Antitoxin	N/A
SokE/HokE	I	Toxin	N/A

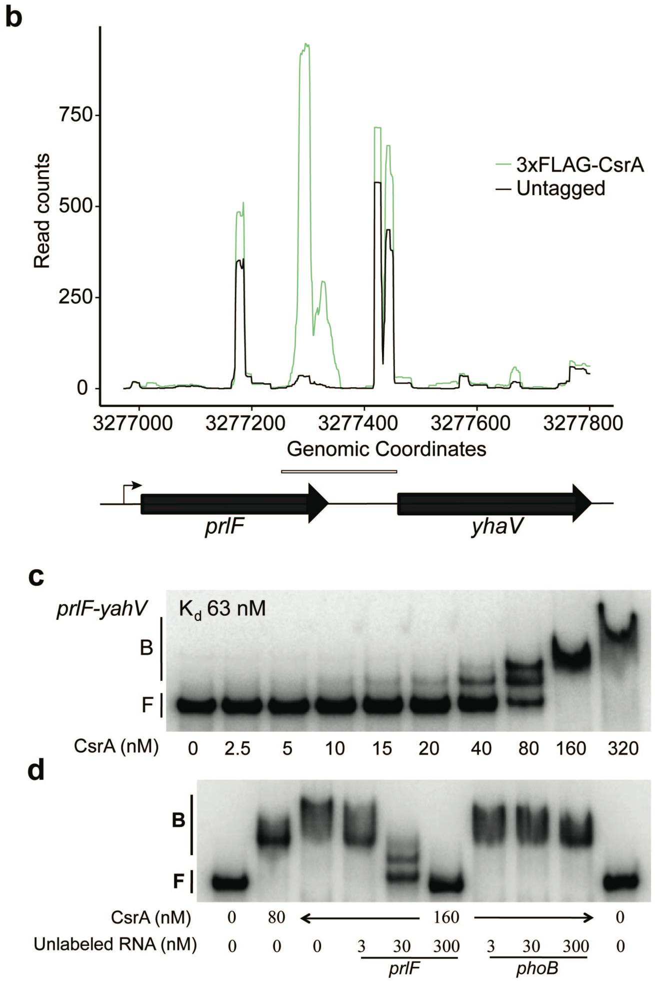

e

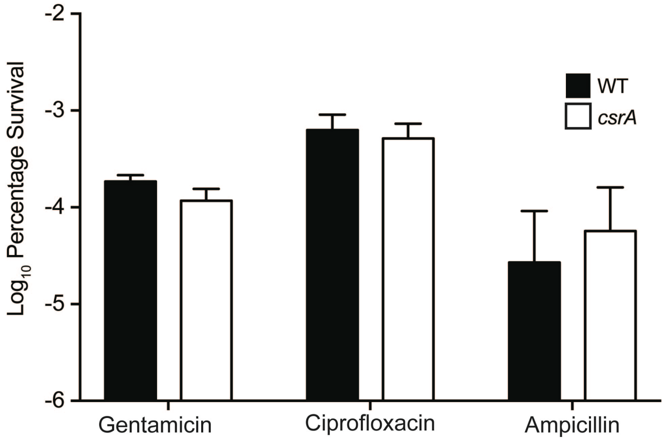

Supplementary Figure 15: CsrA binds to RNAs encoding toxin-antitoxin (TA) systems but does not affect persistence during mid-exponential growth in LB

a. Table summarizing information about the 7 TA systems identified in CLIP-seq analysis, including the type of TA system[10], the position of the peak relative to the toxin/antitoxin genes, and the effect of CsrA on expression. b. 3xFLAG-CsrA and untagged control CLIP-seq read counts in the *prlF-yahV* locus. Position of the Electrophoretic mobility shift assay (EMSA) probe is indicated with a box below the plot. c-d. EMSA showing interaction of CsrA with *prlF-yahV* intergenic region (from -84 to +121 relative to the *yahV* start codon) and competition assay showing the specificity of interaction. F, free RNA; B, bound RNA. Whereas unlabeled *prlF-yahV* RNA was an effective competitor, the non-specific competitor *phoB* RNA was not. e. As TA systems have been associated with changes in persistence[11], we assessed the persistence of the wild type and *csrA* mutant strains after antibiotic treatment. Log percent survival of wild type and *csrA* mutant strains after 4 hr exposure to 10 µg/ml gentamicin, 1 µg/ml ciprofloxacin, or 100 µg/ml ampicillin, using methods described previously[11].

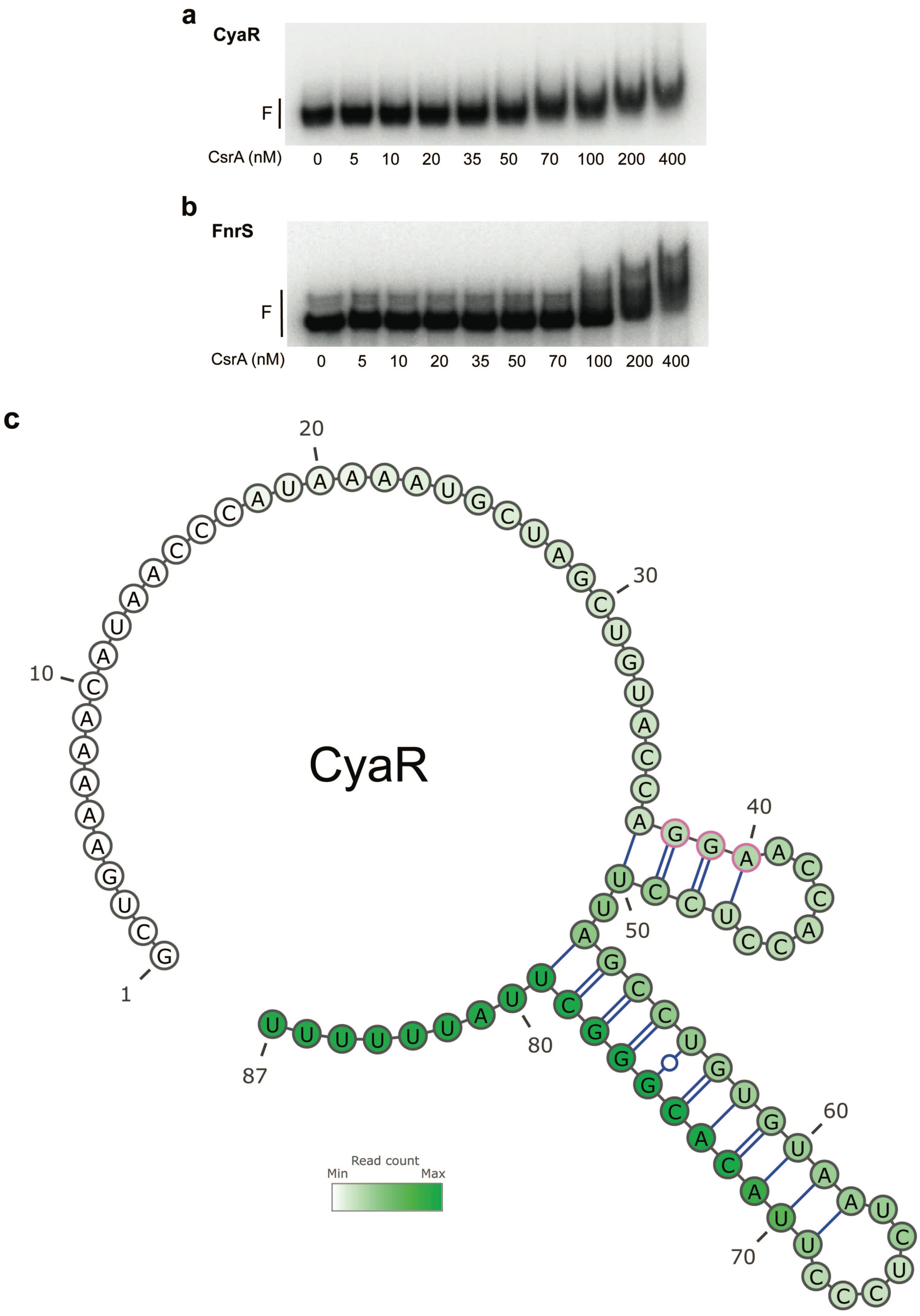
a
CyaR
F
CsrA (nM)
0 5 10 20 35 50 70 100 200 400
b
FnrS
F
CsrA (nM)
0 5 10 20 35 50 70 100 200 400
c
CyaR
1
10
20
30
40
50
60
70
80
87
Read count
Min
Max

d

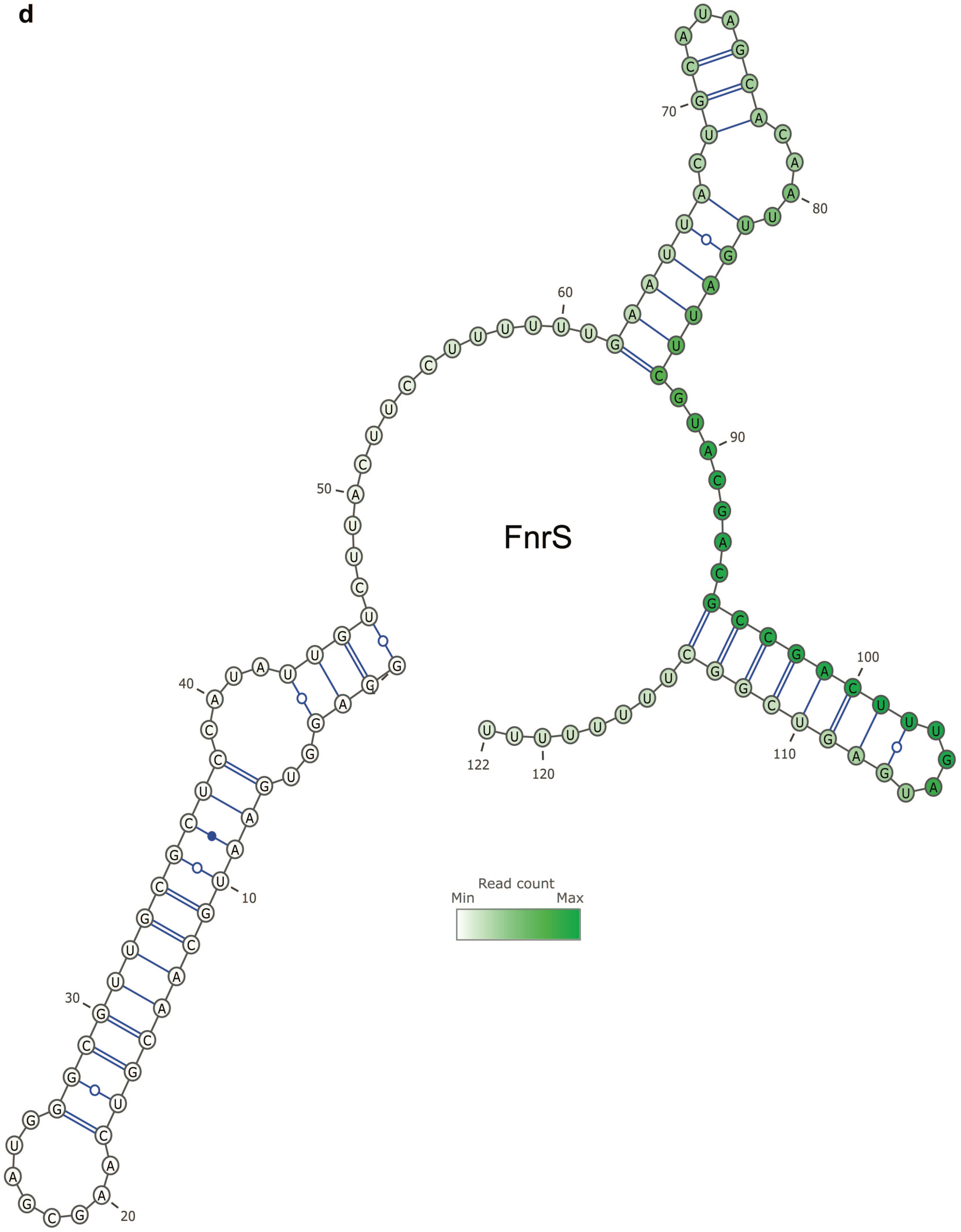

Supplementary Figure 16: CsrA alone does not interact with high affinity to CyaR or FnrS sRNAs *in vitro*

a. Electrophoretic mobility shift assays (EMSA) of CsrA-CyaR interaction. b. EMSA of CsrA-FnrS interaction. c. CyaR[12] and FnrS[13] structures with CLIP-seq reads from all 5 replicates overlaid in green. The GGA motif in CyaR is outlined in pink.

a

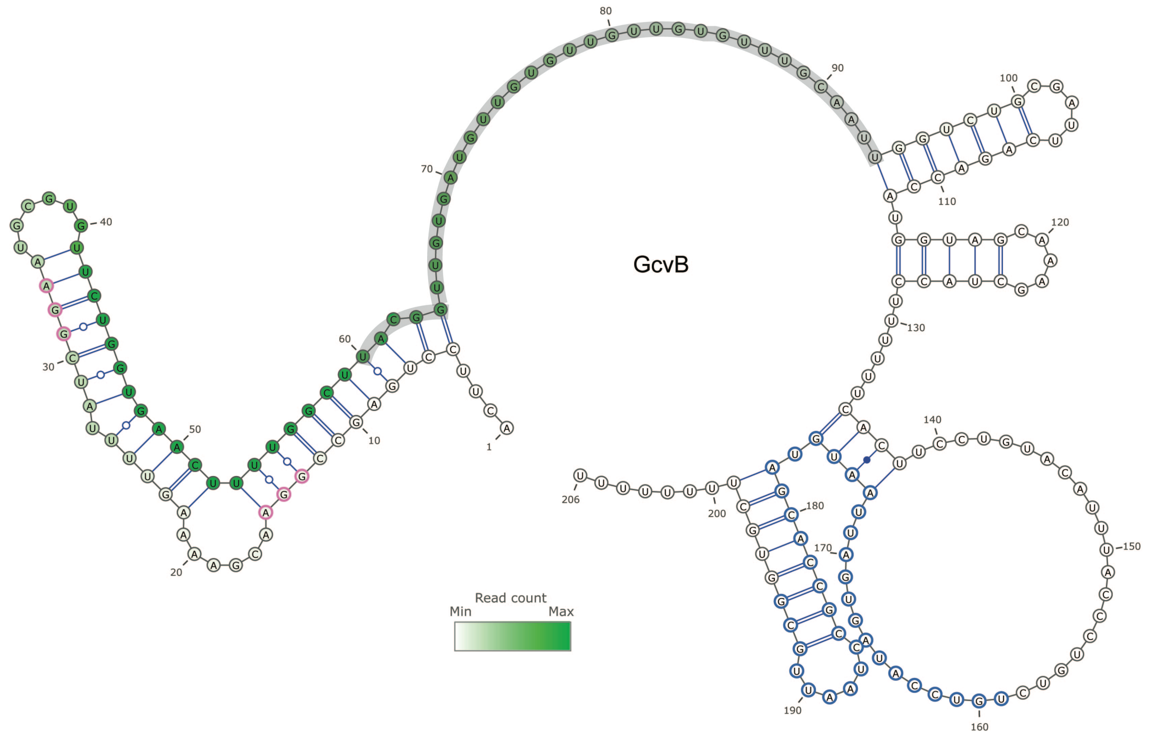

b

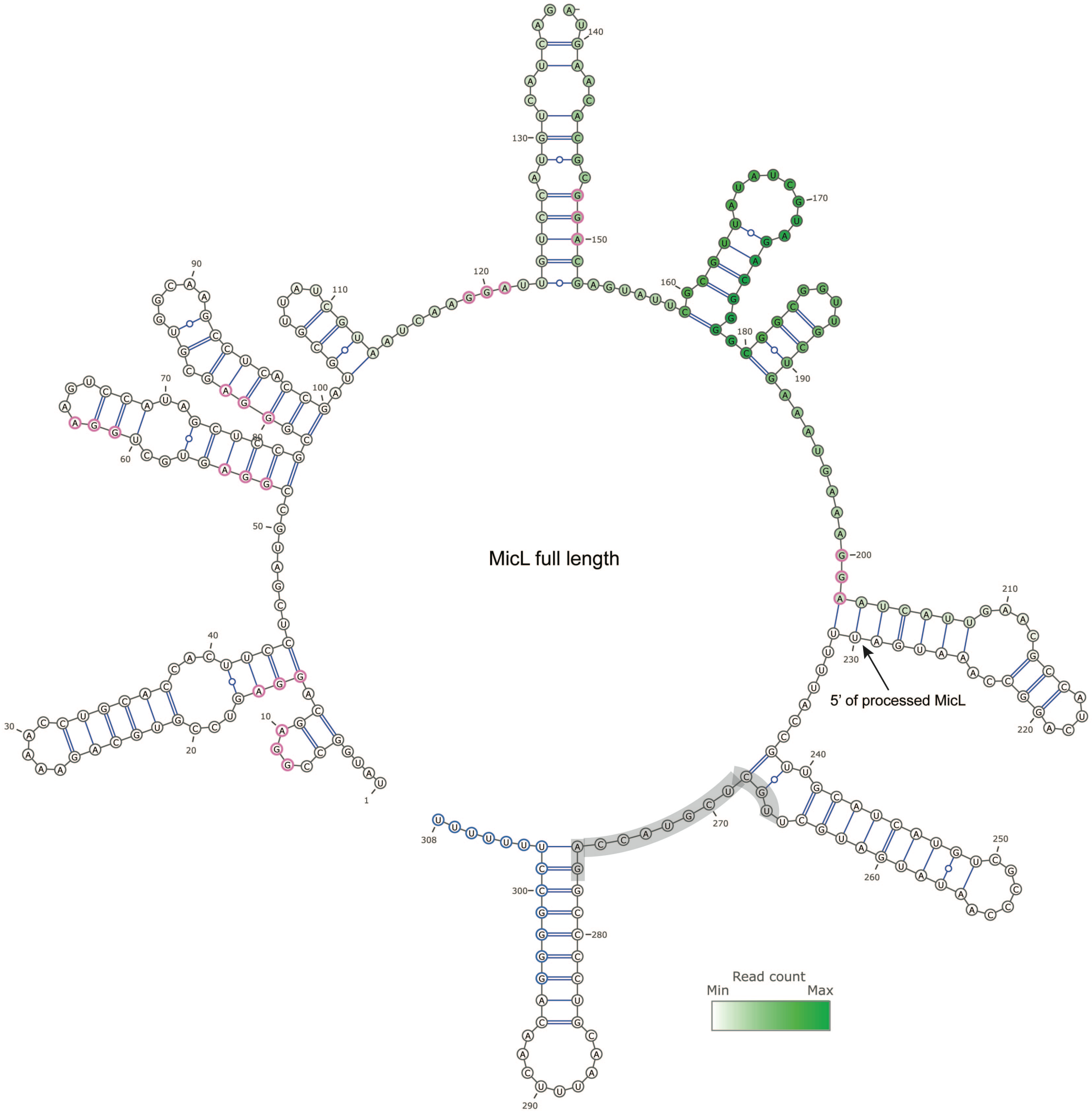

Supplementary Figure 17: Annotated structures of MicL and GcvB
a. Previously published GcvB[14] and b. mFold[15] prediction of full length MicL structures with CLIP-seq reads from all 5 replicates overlaid in green. Putative Hfq binding sites outlined in blue were predicted using the consensus Hfq binding motif identified in *Salmonella*[4]. GGA motifs are outlined in pink. Regions involved in base pairing with mRNA targets in are outlined in grey for MicL[16] and GcvB[14].

a

b

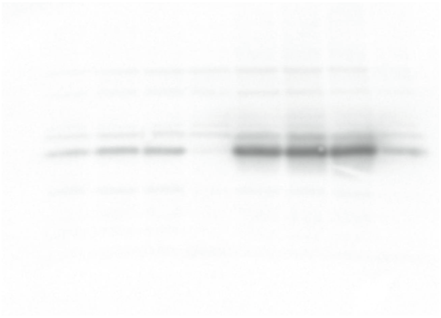

Supplementary Figure 18: 3xFLAG-FtnB and RpoB Western Blot
Raw exposure of a. RpoB and b. 3FLAG-FtnB Western blot from Figure 6.

Supplementary References

1. Kulkarni, P. R. *et al.* A sequence-based approach for prediction of CsrA/RsmA targets in bacteria with experimental validation in *Pseudomonas aeruginosa*. *Nucleic Acids Res.* **42,** 6811–25 (2014).
2. Edwards, A. N. *et al.* Circuitry linking the Csr and stringent response global regulatory systems. *Mol. Microbiol.* **80,** 1561–80 (2011).
3. Sowa, S. W. *et al.* Integrative FourD omics approach profiles the target network of the carbon storage regulatory system. *Nucleic Acids Res.* **45**, 1673-1686 (2017).
4. Holmqvist, E. *et al.* Global RNA recognition patterns of post-transcriptional regulators Hfq and CsrA revealed by UV crosslinking *in vivo*. *EMBO J.* **35,** 991–1011 (2016).
5. Wang, X. *et al.* CsrA post-transcriptionally represses *pgaABCD*, responsible for synthesis of a biofilm polysaccharide adhesin of Escherichia coli. *Mol. Microbiol.* **56,** 1648–63 (2005).
6. Figueroa-Bossi, N. *et al.* RNA remodeling by bacterial global regulator CsrA promotes Rho-dependent transcription termination. *Genes Dev.* **28,** 1239–51 (2014).
7. Thomason, M. K. *et al.* Global Transcriptional Start Site Mapping Using Differential RNA Sequencing Reveals Novel Antisense RNAs in *Escherichia coli*. *J. Bacteriol.* **197,** 18–28 (2015).
8. Dubey, A. K. *et al.* CsrA regulates translation of the *Escherichia coli* carbon starvation gene, *cstA*, by blocking ribosome access to the *cstA* transcript.

J. Bacteriol. **185,** 4450–60 (2003).
9. Gama-Castro, S. *et al.* RegulonDB version 9.0: high-level integration of gene regulation, coexpression, motif clustering and beyond. *Nucleic Acids Res.* **44,** D133–D143 (2016).
10. Page, R. & Peti, W. Toxin-antitoxin systems in bacterial growth arrest and persistence. *Nat Chem Biol* **12,** 208–214 (2016).
11. Maisonneuve, E., Shakespeare, L. J., Girke Jørgensen, M. & Gerdes, K. Bacterial persistence by RNA endonucleases. *Proc. Natl. Acad. Sci.* **108**,13206-11(2011).
12. Papenfort, K. *et al.* Systematic deletion of *Salmonella* small RNA genes identifies CyaR, a conserved CRP-dependent riboregulator of OmpX synthesis. *Mol. Microbiol.* **68,** 890–906 (2008).
13. Durand, S. & Storz, G. Reprogramming of anaerobic metabolism by the FnrS small RNA. *Mol. Microbiol.* **75,** 1215–1231 (2010).
14. Sharma, C. M. *et al.* Pervasive post-transcriptional control of genes involved in amino acid metabolism by the Hfq-dependent GcvB small RNA. *Mol. Microbiol.* **81,** 1144–1165 (2011).
15. Zuker, M. Mfold web server for nucleic acid folding and hybridization prediction. *Nucleic Acids Res.* **31,** 3406–3415 (2003).
16. Guo, M. S. *et al.* MicL, a new σ^E-dependent sRNA, combats envelope stress by repressing synthesis of Lpp, the major outer membrane lipoprotein. *Genes Dev.* **28,** 1620–1634 (2014).
