# Supplementary material for: Global role of the bacterial post-transcriptional regulator CsrA revealed by integrated transcriptomics
Source: Nat Commun. 2017 Nov 17;8:1596. doi: 10.1038/s41467-017-01613-1 (PMC5694010; doi:10.1038/s41467-017-01613-1)
Supplement: Supplementary file 2 — Description of Additional Supplementary Files [file 41467_2017_1613_MOESM2_ESM.pdf]

Description of Additional Supplementary Files

File Name: Supplementary Data 1
Description: CsrA CLIP-seq data

File Name: Supplementary Data 2
Description: Ribosome profiling and RNA-seq data

File Name: Supplementary Data 3
Description: Full DAVID functional enrichment data for genes identified in CLIP-seq

File Name: Supplementary Data 4
Description: Full DAVID functional enrichment data for genes identified in CLIP-seq or differentially expressed in RNA abundance, translation, translation efficiency, and/or RNA stability analyses

File Name: Supplementary Data 5
Description: Full DAVID functional enrichment data for genes identified in CLIP-seq and differentially expressed in RNA abundance, translation, translation efficiency, and/or RNA stability analyses

File Name: Supplementary Data 6
Description: Genes encoding transporters that are regulated by CsrA

File Name: Supplementary Data 7
Description: Genes encoding regulators that are regulated by CsrA

File Name: Supplementary Data 8
Description: sRNAs that interact with CsrA and their assocaited transcriptional regulators and mRNA targets

File Name: Supplementary Data 9
Description: Bacterial Strains

File Name: Supplementary Data 10
Description: Oligonucleotides

File Name: Supplementary Data 11
Description: Summary of sequencing read statistics
